# Influence of the Solvent on the Stability of Aminopurine Tautomers and Properties of the Amino Group

**DOI:** 10.3390/molecules28072993

**Published:** 2023-03-27

**Authors:** Anna Jezuita, Paweł A. Wieczorkiewicz, Tadeusz M. Krygowski, Halina Szatylowicz

**Affiliations:** 1 Faculty of Science and Technology, Jan Dlugosz University in Czestochowa, Al. Armii Krajowej 13/15, 42-200 Czestochowa, Poland; 2 Faculty of Chemistry, Warsaw University of Technology, Noakowskiego 3, 00-664 Warsaw, Poland; 3 Department of Chemistry, Warsaw University, Pasteura 1, 02-093 Warsaw, Poland

**Keywords:** purine, amino group, solvent effect, substituent effect, cSAR, HOMA

## Abstract

Amino derivatives of purine (2-, 6-, 8-, and N-NH_2_) have found many applications in biochemistry. This paper presents the results of a systematic computational study of the substituent and solvent effects in these systems. The issues considered are the electron-donating properties of NH_2_, its geometry, π-electron delocalization in purine rings and tautomeric stability. Calculations were performed in ten environments, with 1 < ε < 109, using the polarizable continuum model of solvation. Electron-donating properties were quantitatively described by cSAR (charge of the substituent active region) parameter and π-electron delocalization by using the HOMA (harmonic oscillator model of aromaticity) index. In aminopurines, NH_2_ proximity interactions depend on its position and the tautomer. The results show that they are the main factor determining how solvation affects the electron-donating strength and geometry of NH_2_. Proximity with the NH∙∙∙HN repulsive interaction between the NH_2_ and endocyclic NH group results in stronger solvent effects than the proximity with two attractive NH∙∙∙N interactions. The effect of amino and nitro (previously studied) substitution on aromaticity was compared; these two groups have, in most cases, the opposite effect, with the largest being in N1H and N3H purine tautomers. The amino group has a smaller effect on the tautomeric preferences of purine than the nitro group. Only in 8-aminopurine do tautomeric preferences change: N7H is more stable than N9H in H_2_O.

## 1. Introduction

Purine derivatives are important in many biological processes. They are, among others, components of nucleic acids (e.g., adenine and guanine) and many co-factors recognized by proteins. Apart from them, adenine (6-aminopurine) is a building block of energy storage units in living cells, ATP and ADP (adenosine 5′-triphosphate and diphosphate, respectively), of the second intracellular messenger cAMP (3′-5′-cyclic adenosine monophosphate) and of coenzyme NAD^+^ (nicotinamide adenine dinucleotide), which is involved in redox reactions within many metabolic pathways [1,2]. Furthermore, several well-known alkaloids, such as theophylline, theobromine, and caffeine, are purine derivatives. Purine derivatives exhibit diverse biological activity and are components of some medicines [3,4]. 

**Scheme 1:**
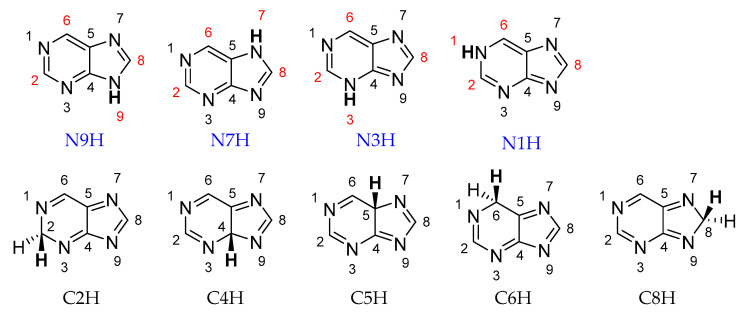
Structures of nine prototropic purine tautomers. Mono-NH_2_-substituted derivatives of four N tautomers are the subject of this study. Substitution positions (C2-, C6-, C8-, and N-) are marked in red.

Purine can exist in the form of nine prototropic tautomers: four NH tautomers with the labile proton at the N atom (N1H, N3H, N7H, or N9H) and five CH tautomers with the labile proton at the C atom (C2H, C4H, C5H, C6H, or C8H; see Scheme 1). 

The NH forms are much more stable, with a difference between the least stable NH form and the most stable CH form of 27 kcal/mol. The amount of individual tautomers in a mixture strongly depends on the environment [5,6]. In both the gas phase (GP) and the aqueous phase, the N9H tautomer is the most stable, with energy of 4.0 (GP) and 0.2 (H_2_O) kcal/mol below the N7H tautomer, according to calculations [5]. Due to this small energy difference in polar solvents, the coexistence of N9H and N7H tautomers in similar amounts is observed in experimental studies (UV [7], NMR [8], and Raman spectra [9]), while, according to the IR matrix isolation study, in the gas phase, the N9H tautomer highly dominates [10,11]. Oxidation or reduction of purine also affects the tautomeric equilibrium. For oxidized species, the N1H tautomer is preferred over N9H. Thus, it clearly indicates that the purine structure is sensitive to the environment and electron-transfer reactions [5]. Other factors influencing the purine tautomerism are the presence of substituent(s) [12,13] and metal complexation [8,14,15].

The unique reactivity of purine allows its structure to be modified in many ways. Positions 2, 6, and 8 are susceptible to attack by nucleophiles, whereas 3 and 7 by electrophiles [16]. Both carbon and nitrogen can be used as anchoring points for attaching various types of functional groups, such as dyes or binding molecules. For nucleosides/nucleotides of purine, C6 is the most common carbon anchoring position, while nitrogen positions are N1 and N7. Different strategies used in the modification of purine nucleosides and nucleotides are presented in the review by Kore et al. [17]. The modification or substitution at C2, C6, C8, or N positions of purine allows to acquire specific properties and functions of the molecule. For example, the fluorination of the C2, C6, and C8 position in purines and purine nucleosides has been studied due to the potential antitumor and antiviral activities of such compounds [18]; examples of anticancer drugs are 6-mercaptopurine [4], cladribine and fludarabine [19]. Moreover, the synthetic variability is needed for implementing drug-like properties [20] and selective protein binding [21] into the purine skeleton. Substituted purines have been shown to form hydrophobic contacts, including π-interactions, and various pairs and triplets of hydrogen bonding, which supports enzyme molecular recognition and/or inhibitory activity [22,23]. Some structurally modified bases are being studied as genetic probes for the study of higher eukaryotic organisms. The adenine analog 2-aminopurine (2AP) is a mutagen which mimics the natural base [24,25]. It has good qualities as a probe: The DNA structure is minimally perturbed by the replacement of adenine by 2-aminopurine; the latter has high fluorescence quantum yield in contrast to the natural nucleic acid bases [26]. It is also used in mutagenesis research, as it forms base pairs with both thymine and cytosine residues. The incorporation of 2AP into the nucleic acids of *E. coli* and other eukaryotic organisms has been well studied [27,28]. The presence of the amino group in the C2 position of purine and the hydroxyl amino group at the C6 position gives one of the most potent mutagen-AHAs (2-amino-6-hydroxylaminopurine); it easily replaces adenine and causes mutations by mispairing with cytosine, being active at relatively low concentrations [29]. In turn, 8-substituted purines have been studied as, for example, kinase inhibitors [30]. The importance of 8-substituted purine derivatives in nucleoside and nucleotide chemistry is also significant. For example, some S-substituted 8-sulfanylpurine derivatives exhibit important biological activities; 8-(benzylsulfanyl)purines are known as irreversible inhibitors of xanthine oxidase [31], and 8[(phenoxyalkyl)sulfanyl]adenosines have hypolipidemic activity [32]. Similarly, N9-substituted analogs of natural purine nucleosides have been widely studied due to their biological activity [33]. N7-nucleoside analogs have been studied less frequently [34], often in the context of N7/N9-glycosyl transfer [35,36]. In addition, purine analogs with the N3 nitrogen replaced by another group (3-deazapurines) are also being studied; such an exchange is biologically important, and alters interactions with proteins, water, and other molecules (see [37] and references therein).

As already mentioned above, the solvent affects the stability of purine tautomers. The same applies to the properties of their derivatives. For example, the solvent effects on the stability of tautomers and π-electron delocalization in 6-amino derivative of purine, adenine, have already been computationally studied by Raczyńska and Makowski [38], taking into account all 23 tautomers. The environment also plays a significant role in the emissive properties of 2-aminopurine: its fluorescence yield increases dramatically with solvent polarity [26]. However, it should be emphasized that most of the theoretical research concerning the nucleic acid base derivatives have been performed in the gas phase [39,40], while theoretical studies on the solvated systems are still limited. Continuum solvation models [41,42,43] are mainly used to account for solvent effects.

The substituent effect (SE) is one of the most useful terms in organic chemistry, describing how group rearrangements or substituent changes in a molecule affect its chemical, physical, and biochemical properties. For its description, substituent constants, σ, are mainly used, which were obtained from the dissociation constants of unsubstituted and *p-/m*-substituted benzoic acids according to the Hammett equations [44,45]. The σ values characterize the electron-withdrawing/donating properties of the substituents. However, the properties of the substituents depend on the character of the reaction center and the nature of intramolecular interactions, as is reflected in many different scales of substituent constants that have been introduced [46,47], for example, different values of substituent constant for the *para* and *meta* positions. Undoubtedly, the use of quantum chemistry methods turned out to be very helpful in the study of the substituent effect. This resulted in the introduction of many concepts (proposed and tested) to its description, for example, MESP (molecular electrostatic potentials) [48], SESE (substituent effect stabilization energy) [49], and cSAR (charge of the substituent active region) [50,51]. The latter substituent effect descriptor seems to be the simplest and is very useful. It can be used to characterize the properties of substituents regardless of the system under consideration, both in the gas phase and in any solvent. Thus, cSAR allows for the comparison of changes in the electronic properties of the substituent (X) and the reaction center (Y) on the same scale. It should be emphasized that the substituent effect can be considered from different points of view, e.g., the classical and reverse substituent effect. For systems presented as X-R-Y (R is a transmitting moiety), the classical substituent effect describes the influence of X on chemical or physicochemical properties of the Y reaction site. Its strength is described by the reaction constant, *ρ*, of the Hammett equation: *P* = *ρ*∙σ + *b*, where *P* is some physicochemical property on which a series of substituents, each of them described by σ, is acting. The reverse substituent effect describes changes in the properties of the substituent X depending on the position in R or the nature of reaction site Y [52]; in other words, it is the effective σ in a given system. Further aspects of the substituent effect are the influence of X on the properties of the transmitting moiety, R, and the effect of the substituent X on the structural fragments of reaction site Y, leading to their mutual correlations [53].

Generally, changes in the strength of substituent effects and substituent properties can result from various factors, e.g., intra- and intermolecular interactions [54] or solvation [55]. An interesting example of the influence of intramolecular interactions on the properties of a substituent is when steric or attractive interactions between neighboring groups cause the rotation of one of them. For example, the rotation of the nitro group, as observed in some compounds [56,57], can decrease the strength of the substituent effect by ~45% in the case of 90° rotation [58].

Recent research on nitro derivatives of purine [59], using the polarizable continuum model (PCM), revealed that solvation generally increases the electron-withdrawing properties of the NO_2_ group. The extent of this increase depends on the through-space proximity interactions experienced by the group—the largest changes were observed in derivatives, in which NO_2_ participated in two repulsive NO···N interactions. Moreover, both the nitro substitution and the solvent have been shown to have a substantial effect on the tautomeric equilibrium. The C6-NO_2_ N7H tautomer (Scheme 1 with X = NO_2_) is more stable than the N9H tautomer in all solvents considered (by about 4 kcal/mol in the gas phase), while the C2-substituted N7H tautomer becomes slightly more stable than the N9H form in more polar solvents. In C8-substituted systems, the least stable N-tautomer of the unsubstituted purine, N1H, becomes the most stable, but the differences between energies of the N9H, N7H, and N1H tautomers are lower than 1 kcal/mol. Interestingly, in C8-nitroadenine, the most stable tautomer in water solution is 3H [60]; thus, it is possible to tune the tautomeric properties of purine derivatives by substitution.

In this paper, we show how the NH_2_ substitution at the C (C2, C6, or C8) or N (N1, N3, N7, or N9) positions of purine affects the electron-donating properties of the amino group, its geometry, the π-electron structure of the purine rings, and the tautomeric preferences of aminopurines (Scheme 1). For all studied systems, DFT calculations were performed in nine solvents (PCM, 1 < ɛ < 109) and in the gas phase in order to describe the solvent effects in different environments in which biological processes take place.

## 2. Results

The presentation of the results is divided into four parts concerning: electronic properties of the amino group, geometry and proximity interactions, π-electron delocalization in five- and six-membered rings of purine, and the stability of tautomers of aminopurines. All obtained characteristics of the studied systems are collected in Supplementary Tables S2–S11. In this section, the shortened tautomer notation is used, i.e., 9H, 7H, 3H, and 1H, instead of N9H, N7H, N3H, and N1H, respectively.

**Scheme 2:**
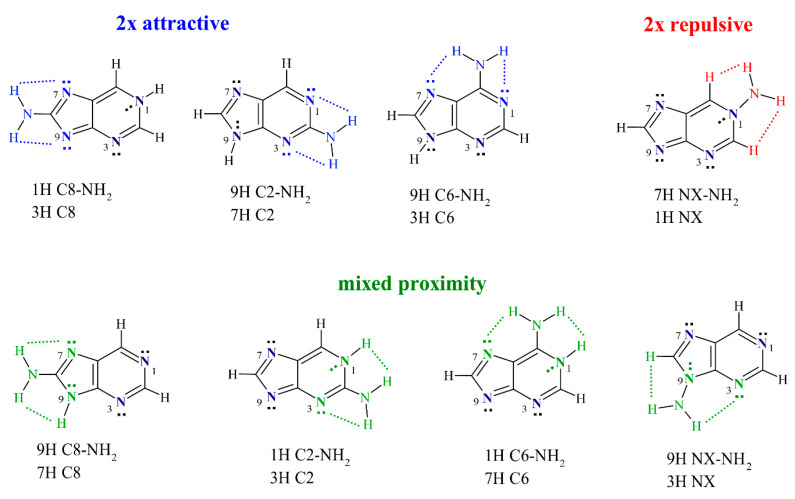
Classification of the proximity interactions into three types used throughout this paper, along with the systems belonging to each type. The presented color-coding scheme is maintained in all Figures.

First, it should be mentioned that the proximity interactions in nucleic acid base derivatives can be somewhat complicated due to the existence of the endocyclic N or NH groups in various positions. In these systems, the NH_2_ group can be a hydrogen bond donor and can engage in attractive interactions with endocyclic N atoms (NH···N). Moreover, when the NH group is present in the proximity, a repulsive (NH···HN) interaction occurs. In the N-NH_2_ substitution, the amino group can also interact with neighboring CH groups, which (in this case) is also a repulsive interaction (NH···HC). Therefore, throughout this paper, the proximity effects will be classified into three types: attractive, mixed, and repulsive, shown in Scheme 2. In the 2× attractive proximity, NH_2_ engages in two attractive interactions (2× NH···N). In the mixed proximity, NH_2_ experiences one attractive (NH···N) and one repulsive interaction (NH···HN or NH···HC), whereas, in the 2× repulsive proximity, only two repulsive (NH···HC) interactions are present.

### 2.1. Electronic Properties of the Amino Group

In order to evaluate and compare the electron-donating strength of the NH_2_ group in studied aminopurines, the cSAR parameter of the NH_2_ group was calculated for each system. Taking into account all systems, the values of cSAR(NH_2_) range from 0.133 to 0.375. Thus, the electron-donating power of the NH_2_ group may change by as much as 0.242e due to the factors considered in this study: substitution position, tautomeric form of aminopurine, and solvent effects. For comparison, the cSAR(NO_2_) values of the NO_2_ group in nitropurines indicate that this group changes its electron-withdrawing ability by 0.142e, due to similar factors [59]. Thus, for substituted purine derivatives, the reverse substituent effect of the amino group is much greater than that of the nitro group.

**Figure 1:**
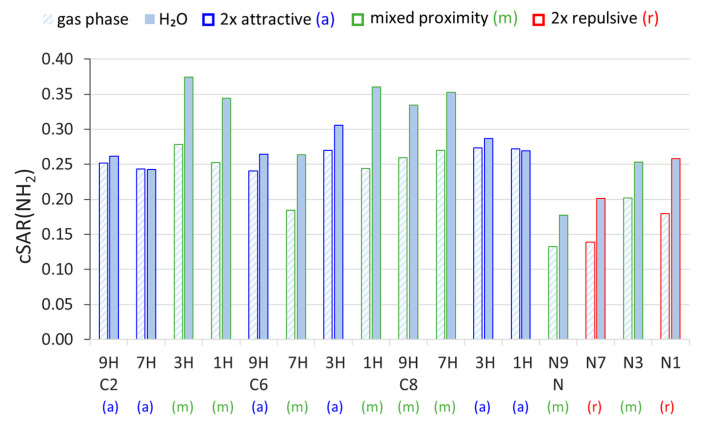
Values of cSAR(NH_2_) in 9H, 7H, 3H, and 1H tautomeric forms of C- and N-NH_2_ derivatives of purine, calculated in the gaseous and aqueous phases. The color coding of each bar indicates the proximity interactions present in each system.

The cSAR values of the amino group, cSAR(NH_2_), presented in Figure 1, indicate its electron-donating properties in each system. First, it can be noticed that the values in the N-NH_2_ systems are, on average, lower than in the C NH_2_. Moreover, when comparing the substitution positions and tautomeric forms, we can see that the properties of the NH_2_ group differ. Considering the gas phase, for each substitution position, the NH_2_ group is slightly more electron donating in 1H and 3H tautomeric forms of aminopurine (NH group in the six-membered ring) than in 7H and 9H (NH group in the five-membered ring). No statistically significant differences were found between cSAR(NH_2_) values in the gas phase for different proximity types (*t*-test). The differences between the cSAR(NH_2_) values calculated in the gas and water phases indicate that solvation is another factor affecting the ED properties of the amino group. An increase in the polarity of the solvent increases its ED properties, except for the two systems with the weakest solvent effects: C2 7H and C8 1H. Additionally, the strength of the solvent effects (extent of changes in the substituent properties induced by solvation) depends on the proximity effects experienced by the NH_2_ group. Systems with at least one repulsive interaction of the NH_2_ (mixed and 2× repulsive proximity) experience much stronger solvent effects than the systems with two attractive interactions. In N-NH_2_ systems, the difference in cSAR(NH_2_) between the gas phase and H_2_O is slightly higher in 2× repulsive systems than in the mixed ones. Among them, the least sensitive position to the solvent effect is the N9 position–the place of bonding between saccharide and nucleobase via the N-glycosidic bond. Differences in cSAR(NH_2_) variability due to solvation between the two proximity effects types in C-NH_2_ systems are statistically significant (*t*-test; *p*-value = 2.27 × 10^−6^); only two values per proximity type in N-NH_2_ systems do not allow us to perform such an analysis.

It should be mentioned that the reciprocal of the solvent dielectric constant (1/ε, Table 1) and cSAR(NH_2_) can be correlated linearly with, in most cases, large determination coefficients, *R*^2^. Therefore, the values of slopes (*a*) of the obtained cSAR(NH_2_) = *a*∙(1/ε) + *b* relations allow to quantitatively compare the sensitivities to the solvent effect of different aminopurines. Appropriate data for all studied systems are presented in Table 2. Additionally, the linear character of the cSAR(NH_2_) vs. 1/ε relation means that most changes in cSAR(NH_2_) occur in the solvents with ε < 10, and only minor changes occur in more polar solvents (Supplementary Table S2). Two systems with small *R*^2^ values, C2 7H and C8 1H, are the only ones in which the electron-donating properties slightly decrease in polar solvents, as evidenced by small and negative Δ (positive a) values (Table 2). Clearly, the greatest differences between the sensitivities occur in the C2- and C8-NH_2_ purines, which are tautomers with mixed and 2× attractive proximity. In these cases, mixed proximity systems are, on average, 20 and 16 times more sensitive to the solvent effect, respectively, whereas, in C6-NH_2_ systems (adenine), only a 3.3-fold difference between these two proximity types is computed. Regarding the N-NH_2_ systems, those with 2× repulsive proximity experience slightly stronger solvent effects than mixed ones. In this case, the ratio between average values of *a* for the two 2× repulsive and the two mixed tautomers is 1.5. Thus, it appears that the existence of repulsive interactions increases the sensitivity of the electronic properties of the NH_2_ group to the solvent effect; moreover, two repulsive interactions further increase it.

In the study, which concerned the nitro derivatives of purine [59], a higher sensitivity to the solvent effect was found in systems with 2× repulsive proximity (2× NO∙∙∙N) contacts than with the mixed one (1× NO∙∙∙N and 1× NO∙∙∙HN). The sensitivity in tautomers with 2× repulsive proximity was, on average, 3.2 times higher in C8 derivatives, 3.6 in C6, and 5.6 in C2 than in mixed-type systems.

### 2.2. Geometric Parameters of the Amino Group

**Scheme 3:**
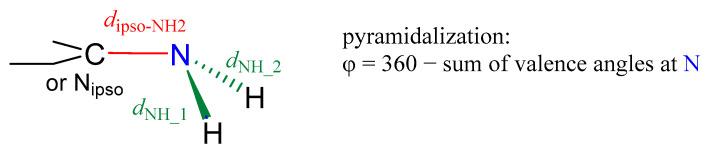
Geometric parameters of the amino group.

**Figure 2:**
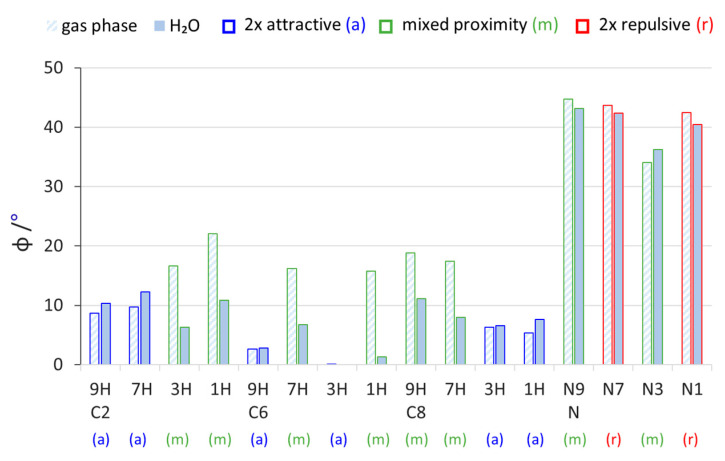
Values of pyramidalization of the NH_2_ group (φ = 360° − sum of valence angles at the N atom) in all studied aminopurines calculated in the gas phase and water (H_2_O).

The amino group can be characterized by its structural parameters, such as the bond lengths and angles (Scheme 3). A particularly important parameter for the interactions of the NH_2_ group is its pyramidalization (φ). It can be calculated as 360° minus a sum of all valence angles at the N atom (Figure 2). Larger pyramidalization indicates the greater sp^3^ character of the hybrid orbitals at the N atom and more of a tetrahedral shape for the amino group. Such a group has a highly localized lone electron pair (Lewis basic site), which can be easily accessed by Lewis acidic centers to form an intermolecular interaction. On the other hand, small pyramidalization indicates a more planar shape, where the central N atom is sp^2^ hybridized. Such a shape indicates a substantial delocalization of the lone electron pair onto the rest of the substituted system. Studies of aniline derivatives show that the shape of the NH_2_ group (its pyramidalization) depends on the substituent effects (intramolecular interaction), as well as on intermolecular interaction by H-bonding [54]. For example, a decrease in the pyramidalization of the NH_2_ group may be related to an increase in the π-electron-accepting power of the *para*-substituent (substituent effect quantified by σ_p_^−^) or the formation of a NH···B (B–Lewis base) hydrogen bond by the NH_2_ group. On the contrary, an increase is computed when the NH_2_ group is involved in the N···HB interaction (HB-Brønsted acid).

Comparing the different tautomeric forms of the C2-, C6-, and C8-substituted aminopurines in the gas phase, we can see that the 2× attractive proximity type systems have lower values of pyramidalization than the mixed ones (Figure 2). Thus, the presence of two attractive NH∙∙∙N interactions promotes more of a planar shape for the NH_2_ group. In the C6 3H system, the amino group is completely planar (φ = 0°), while in C6 9H, it is almost planar (φ = 2.7°). This probably comes from the relatively strong NH∙∙∙N interactions in these systems, one of which (with the N7 atom, Scheme 2) has a favorable geometry for the five-membered ring. Interestingly, the solvation effects act oppositely in 2× attractive and mixed-type systems; namely, the values of φ increase in the former and decrease in the latter. The decreases in the mixed-type systems are so substantial that, in some cases, they become more planar than their 2× attractive counterparts for a given substitution. There may be two reasons for this. First, the solvation of polar fragments of molecules—for example the repulsive NH∙∙∙HN interaction between two partially positively charged hydrogen atoms or the attractive NH∙∙∙N interaction between positively charged hydrogen and negatively charged nitrogen—decreases the strength of such interactions (all proximity interactions between partially charged fragments are weakened). Thus, in the polar environment, less planarization coming from NH∙∙∙N interactions and less pyramidalization due to NH∙∙∙HN repulsion is predicted by the calculations. The second reason can be a change in the electronic properties of the NH_2_ group in polar solvents. Relatively large increases in the electron-donating strength of substituents due to solvation in mixed-type systems (Figure 1) suggest an increase in resonance between the NH_2_ and the purine moiety, which increases the sp^2^ character of the hybrid orbital at the N atom and planarizes the amino group. On the contrary, in 2×-attractive-type systems, solvation only slightly increases its electron-donating properties; thus, the changes in pyramidalization should be much smaller. Values of φ in the N-NH_2_ systems are noticeably larger than in the C-NH_2_ systems, probably due to the different character of the NN bond than the CN bond between the NH_2_ and substituted atom of purine.

**Figure 3:**
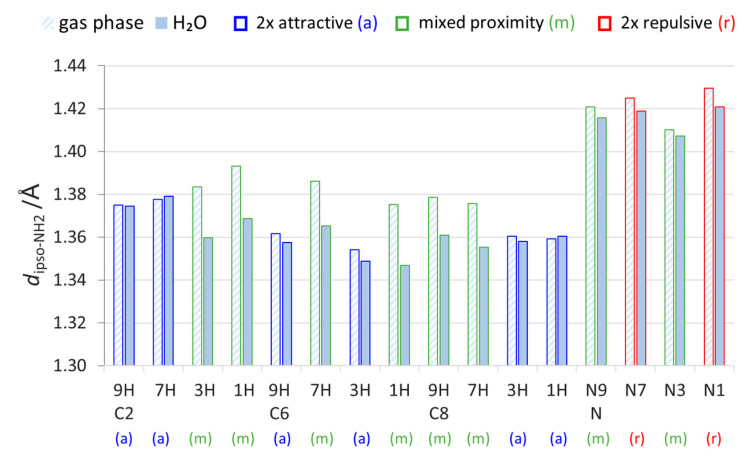
Lengths of bonds connecting the NH_2_ with substituted C or N atom of purine moiety, *d*_ipso-NH2_, in all studied aminopurines calculated in the gas phase and water (H_2_O).

Another parameter worth considering is the bond length between the NH_2_ nitrogen atom and substituted atom of purine (C or N), herein called d_ipso-NH2_ (illustrated in Scheme 3). It indicates the strength of interaction of the NH_2_ group with the substituted system. In the gas phase, the bonds in mixed and 2×-repulsive-type systems for particular substitution are longer than in water, as shown in Figure 3. 

As mentioned earlier, solvation weakens the through-space proximity interactions. Accordingly, in systems with at least one repulsive interaction (mixed and 2× repulsive) that “forces” the bond to be longer in the gas phase, solvation allows it to be shortened. In the solvent environment, *d*_ipso-NH2_ becomes shorter in some mixed-type systems than in 2× attractive systems. The length differences in H_2_O and in the gas phase (Supplementary Table S4) indicate that, in mixed-type systems, the CN bonds are clearly shortened with an increase in solvent polarity (by 0.018–0.029 Å), whereas, in 2× attractive systems, the changes are slight (from −0.005 to +0.002 Å). These differences result from how the weakening of through-space interactions by solvation affects both systems. In mixed-proximity systems, the NH∙∙∙HN interaction in the gas phase seems to be strong enough to dictate the CN bond length; however, solvation of the two positively charged NH hydrogen atoms weakens their repulsion. It follows that, in the polar environment, the CN bond can be shortened. In systems with 2× attractive interactions, there is no steric hindrance for the CN bond to obtain its shorter “optimal” length; hence, solvation does not change its length as much. 

**Figure 4:**
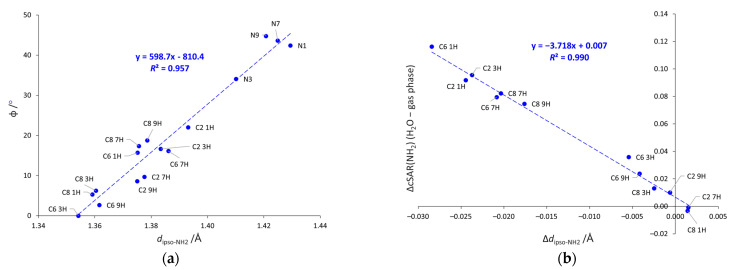
Dependences between (**a**) the pyramidalization, angle φ, and *d*_ipso-NH2_, and (**b**) differences, Δ, in cSAR(NH_2_) and *d*_ipso-NH2_ values calculated in water and in the gas phase (Δ = 0 means that the same values of a given parameter were obtained in water and in gas phase). N-NH_2_ systems not included in (**b**).

Both the geometric parameters discussed above, φ and *d*_ipso-NH2_, are mutually correlated (see Figure 4a). Shorter bonds are present in systems with more planar NH_2_ group, in line with changes in hybridization and the rules of valence bond theory. It is worth noting that the points corresponding to the systems with the NH_2_ group attached to the five-membered ring are grouped above the trendline, whereas the analogous points for the six-membered substituted systems are below it (except C6 1H). This suggests that the NH_2_ group has generally slightly more tetrahedral shape when substituted to the five-membered ring than to the six-membered one. In addition, the strengths of the solvent effects on electronic properties and geometry are also well-correlated, as shown in Figure 4b. The increase in the electron-donating properties of the NH_2_ group due to solvation is closely related to the shortening of the C_ipso-NH2_ bond.

**Figure 5:**
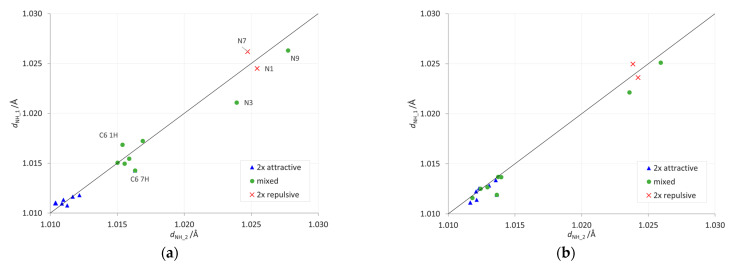
Dependences between the lengths of two NH bonds of the NH_2_ group. The y = x line indicates a situation when both have the same length. Data for (**a**) the gas phase and (**b**) the most polar solvent considered, formamide.

The proximity interactions of the NH_2_ group can be detected by plotting the lengths of its two NH bonds against each other (Figure 5 and Supplementary Table S5). Deviation from the y = x line in such a plot indicates that the group interacts stronger on one side than on the other. Here, the strongest deviations are detected for the gas phase (Figure 5a) in the C6 1H and 7H systems, as well as in the N-NH_2_ systems. Interestingly, the same plot created for the data in formamide (Figure 5b) confirms that solvation weakens repulsive through-space proximity interactions, because the points for mixed-type and 2×-attractive-type systems group together.

Analysis of the proximity effects, using the NCI method, only reveals weak NH∙∙∙N interactions classified as van der Waals interactions in C6 1H, N-NH_2_ 3H, and N-NH_2_ 9H aminopurines (Supplementary Figure S1). Additionally, the AIM analysis detects no bond critical points corresponding to these interactions; thus, they are too weak to be called hydrogen bonds. The reason for that is probably the large distance between N and H atoms, together with the low value of the NH∙∙∙N angle (2.6–2.7 Å and ~100° in systems where the interaction has five-membered ring geometry and 2.4–2.5 Å, ~70° in other (Table S6). On the other hand, the bulkier NO_2_ group can engage in a stronger intramolecular interaction with the endocyclic NH groups in heterocycles [61], which sometimes meet the criteria for the hydrogen-bond formation [62].

### 2.3. π-Electron Delocalization in Aminopurine Tautomers

**Figure 6:**
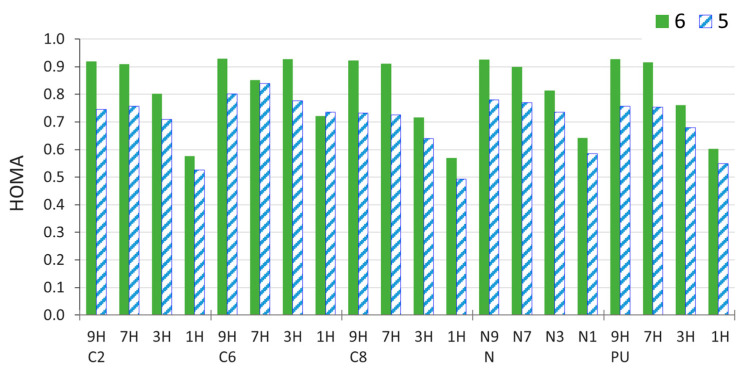
Values of HOMA index for 6- and 5-membered rings of C2-, C6-, C8-, and N-amino-substituted derivatives of purine in four tautomeric forms, calculated for the gas-phase geometry. PU is the values for unsubstituted purine.

One of the aspects of the substituent effect is its influence on the π-electron structure of the transmitting moiety. Before discussing these effects, it is important to mention that, in purine, the π-electron structure depends on the tautomeric form considered. This is because the endocyclic NH group has two 2p_z_ electrons, which can be delocalized within the rings, whereas the endocyclic N atom provides just one π-delocalizable electron. Therefore, 9H and 7H tautomers with the NH group in the five-membered ring consist of imidazole and pyrimidine rings, both of which have 6π delocalizable electrons, and hence are aromatic according to the Hückel 4n + 2 rule. However, in 1H and 3H tautomers with the NH group in the six-membered ring, the six-membered ring contains 7π electrons, while the five-membered ring has 5π electrons. Consequently, the 9H and 7H tautomers of unsubstituted purine have higher aromaticity, as evidenced by aromaticity indices, e.g., HOMA (Figure 6 and Supplementary Table S7).

**Figure 7:**
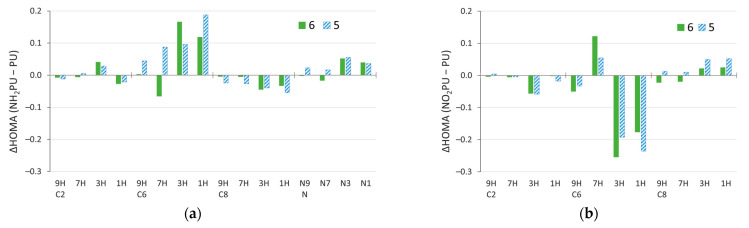
Changes in HOMA index due to substitution, ΔHOMA, calculated by subtracting the value for unsubstituted purine (PU) from the value for substituted purine (NH_2_PU or NO_2_PU). Data for the (**a**) amino and, for comparison, (**b**) nitro derivatives [59].

The NH_2_ group in the C8 and C2 positions reduces the aromaticity of both purine rings in all tautomers, except for the five-membered ring in C2 3H and 7H systems (Figure 7a). However, in the C6 and N-NH_2_ derivatives, the aromaticity of both rings increases, except for the six-membered ring in N-NH_2_ 9H and 7H. Changes in the aromaticity are more pronounced in the 1H and 3H tautomers than in the 7H and 9H ones. Thus, the π-electron structure in “aromatic” tautomers tends to be preserved, whereas, in “nonaromatic” ones, it can change significantly due to the substituent effect. Interestingly, the aromaticity of the six-membered ring in C6 3H (so, the 3H tautomer of adenine) increases so much that its aromaticity is noticeably higher than in the C6 7H tautomer (a decrease of aromaticity of the latter also assists this exchange).

Larger changes in the aromaticity of the 1H and 3H tautomers were also found for the NO_2_ substitution of purine (Figure 7b and Supplementary Table S8). Importantly, the electron-donating NH_2_ and electron-withdrawing NO_2_ groups have the opposite effect on aromaticity in several cases. This is easiest to see in C6 derivatives, where NH_2_ increases aromaticity of the 1H and 3H tautomers substantially (by ~0.1–0.2 HOMA units), while NO_2_ decreases it (by ~0.20–0.25). A similar effect happens for the 9H tautomer, but in 7H, the effects of these two groups are reversed. This is probably due to the distortion of geometry by a relatively strong NH/NO∙∙∙HN interaction, repulsive and attractive, respectively, with a six-membered ring geometry present in these systems. In C8 derivatives, apart from the six-membered ring in the 7H and 9H tautomers, NO_2_ increases aromaticity, whereas the NH_2_ group decreases it.

**Figure 8:**
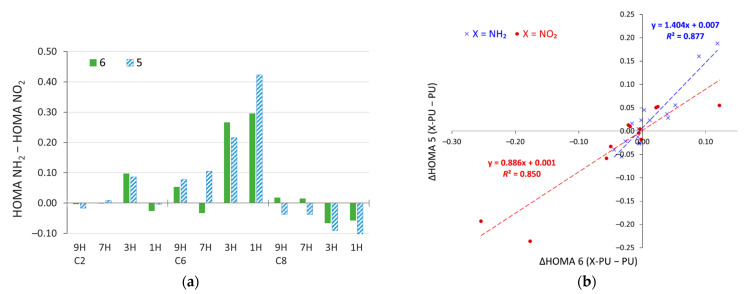
(**a**) Differences in the values of HOMA for amino and nitro derivatives of purine and (**b**) changes in HOMA due to substitution for 5- and 6-membered rings plotted against each other.

The differences between the HOMA values in amino and nitro derivatives of purine are shown in Figure 8a. They are clearly most pronounced for the C6 substitution, but also in the C2 3H and C8 3H and 1H systems. Plotting changes in HOMA due to substitution (with respect to unsubstituted purine) in the five-membered ring against the corresponding value for the six-membered ring (Figure 8b) reveals that, in NH_2_ derivatives, changes in the aromaticity of the five-membered ring are statistically 40% larger than those in the six-membered ring (slope value, *a* = 1.404). Thus, the substituent effect of the NH_2_ group is acting stronger on the five-membered ring. On the other hand, the NO_2_ substitution gives a slope value lower than 1 (*a* = 0.886). Thus, statistically, the substituent effect of the NO_2_ group changes the π-electron structure of the six-membered ring to a greater extent than the five-membered one. The opposite effects for the electron-donating NH_2_ and electron-withdrawing NO_2_ groups most likely come from the fact that, in the “nonaromatic” 1H and 3H tautomers, the 6-membered ring has an excess of π electrons (7), whereas the 5-membered is deficit (5), compared to the 6π electrons needed for the Hückel aromaticity.

**Figure 9:**
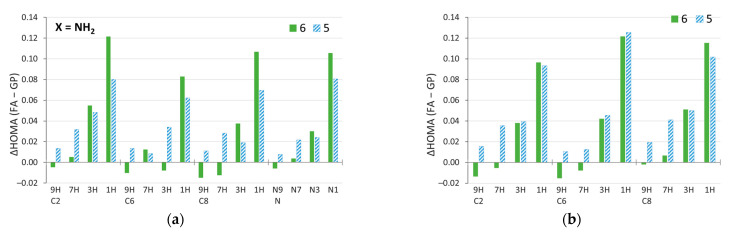
Changes in HOMA index due to solvation ΔHOMA (FA–GP), calculated by subtracting the value in the gas phase from the value in the most polar solvent considered, formamide. Data for the (**a**) amino and, for comparison, (**b**) nitro derivatives [59].

The π-electron structure can also change due to solvation. Figure 9 presents the differences in HOMA aromaticity indices calculated for the geometry in the most polar solvent and in the gas phase. In most cases, aromaticity increases in polar solvents. Moreover, the increases of HOMA are much more pronounced than the decreases; the maximum decrease is 0.015, while the increase can be as high as 0.120. This applies to both NH_2_ (Figure 9a) and NO_2_ (Figure 9b) substitutions. By far, the largest changes occur in the 1H tautomers (least aromatic in the gas phase, Figure 6) for all substitution positions. Moreover, the six-membered rings of the 1H amino derivatives are more sensitive to solvation than the five-membered ones (Figure 9a), and generally the same is true for all amine systems (Supplementary Figure S2a). Nonaromatic 1H and 3H tautomers are more prone to changes in aromaticity due to solvation (Figure 9) and, as shown earlier, by substitution. It should be added that relatively similar results were obtained by using the Harmonic Oscillator Model of Electron Delocalization (HOMED) index [63] (see Supplementary Figures S2b, S3, and S4).

### 2.4. Stability of Aminopurine Tautomers in Various Solvents

**Figure 10:**
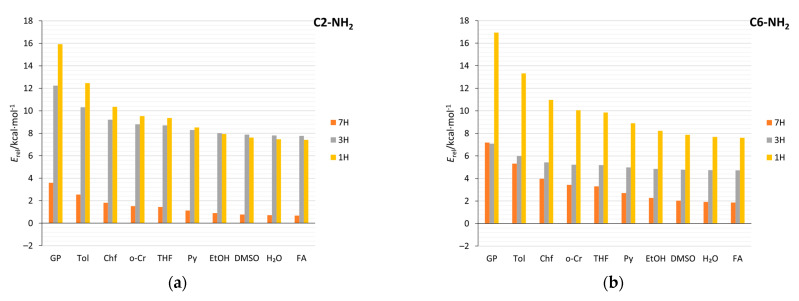

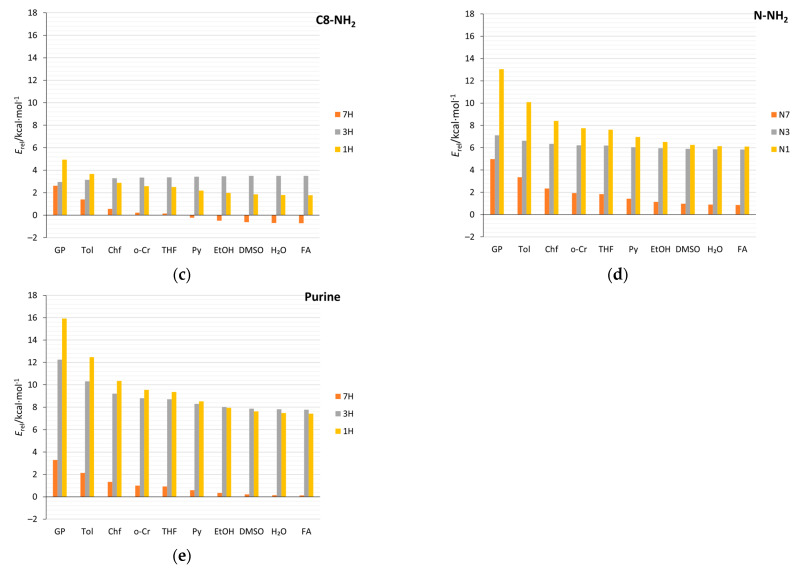
Relative energies of tautomers (*E*_rel_) in various solvents (abbreviations and dielectric constants in Table 1), with respect to the 9H tautomer, for (**a**) C2-NH_2_, (**b**) C6-NH_2_ (**c**) C8-NH_2_ (**d**) N-NH_2_, and (**e**) unsubstituted purines.

Solvation, as well as the substitution of N-heterocyclic compounds, can affect the relative stabilities of tautomers and, in some cases, change the tautomeric preferences [11,64]. Data in Table 3 and Supplementary Table S9 cover both substituent and solvent effects on the relative energy of tautomers (*E*_rel_) in studied aminopurines. For comparison, the relative stabilities of the unsubstituted and nitro-substituted purine tautomers are also included. Regarding the aminopurines, in the gas phase, the 9H tautomer is the most stable for all types of substitutions. For C2-, C8-, and N-NH_2_-substituted purines, the stability decreases in order from the most stable tautomer, 9H, through 7H, 3H, to 1H, similar to the unsubstituted purine tautomers. However, in C8-NH_2_ derivatives, in solvents with ɛ > 10, tautomeric preferences change: The 7H tautomer is more stable than the 9H (Figure 10c). In C2- and C8-NH_2_, like in the unsubstituted purine, solvation highly stabilizes the 1H tautomer, so that it becomes more stable than the 3H. This is not found for the C6- and N-NH_2_ tautomers. In C6 derivatives (adenine), in the gas phase, the 3H tautomer is more stable than the 7H tautomer, and the stability sequence is 9H, 3H, 7H, and 1H, while in water, it changes to 9H, 7H, 3H, and 1H.

In all cases, an increase in the solvent polarity decreases the differences in energy between the most and least stable tautomers. For all amino derivatives, except for the C8-NH_2_ systems, and the unsubstituted purine, this difference decreases by about 50%. As indicated by the ∆ values in Table 3, the relative energies of the 1H tautomer derivatives are the most sensitive to the solvent effect—the greatest stabilizations are predicted. In an aqueous environment, the differences between the energies (*E_rel_*) of the 9H and 7H tautomers are small. This means that both tautomers can coexist in the solution, just as it was computed for unsubstituted purine [5]. In the NO_2_ derivatives, more interesting things happen; for example, the 7H C6-NO_2_ tautomer is much more stable (due to intramolecular hydrogen bonding) than 9H in both the gas phase and water (by more than 4 and 2 kcal/mol, respectively). Additionally, in C8-NO_2_, 1H becomes the most stable tautomer in polar environments due to its exceptional stabilization by the solvent (∆ = −11.6 kcal/mol).

In order to explain why some systems are stabilized better than others, the solvation energies (Supplementary Table S10) and their dependence on 1/ε of solvent were analyzed (Table 4). Considering the tautomers of the C-NH_2_ derivatives, it can be noticed that the substitutions which cause the mixed-type proximity effect have higher dipole moments than the 2× attractive ones. Rather, this is due not to the proximity effects but to the fact that when NH and NH_2_ are close to each other (like in mixed-type systems), the vectors of their contributions to the molecular dipole moment add up in a way that the overall dipole moment increases. This leads to higher solvation energies in mixed-type systems. Additionally, the dipole moments increase with the polarity of the solvent in these systems to a greater extent (Supplementary Table S11), as indicated by Δ*μ*. For this reason, the solvation energies increase more in mixed than in 2× attractive systems, as evidenced by the values of *a* (Table 4). Here, the possible cause might be that cSAR(NH_2_) in mixed-type systems increases substantially due to solvation, and, as the electron-donating properties of NH_2_ increase, this contributes to the overall increase in the polarity of charge distribution in the molecule. Moreover, 1H tautomers have the highest dipole moments and the highest increases of them due to solvation. This is the reason for the predicted largest solvation energies and changes in relative energies (Table 3, Figure 10, and Supplementary Figure S5) for 1H tautomers of unsubstituted, as well as amino-substituted, purine.

## 3. Methodology

The research objects were monosubstituted C- and N-NH_2_ purines in the four most stable tautomeric forms. For studied systems, optimization was carried out without any symmetry constraints (in the gas phase and solution), with the use of the Gaussian09 package [66]. Calculations were performed using the DFT method at B97D3 functional [67], with Dunning’s aug-cc-pVDZ basis set [68]. The choice of this computational level was based on its ability to characterize stacking interactions in adenine dimers, with both the energetic and geometric criteria considered [69]. In the case of the N-NH_2_ substitution, the conformation in which the NH_2_ group is perpendicular to the plane of the purine ring is the most stable (see Supplementary Materials Table S1). In these systems, an optimization constraint was added to forbid the rotation of the NH_2_ group around the N-N bond and to force “coplanar” conformation. This way, the types of proximity effect in the N-NH_2_ systems can be classified along with the C-NH_2_ systems, and the comparison between them is easier. However, the values of energy and dipole moments in the manuscript are given for the most stable (perpendicular) conformation. To confirm that presented structures correspond to the minima on the potential energy surface, the vibrational frequencies were calculated at the same level of theory. No imaginary frequencies were found in the C-NH_2_ systems and N-NH_2_ systems with the perpendicular conformation of the NH_2_; in N-NH_2_ systems with a forced “coplanar” NH_2_ group, one imaginary frequency associated with rotation was found. 

The PCM model of solvation [41] was used to study the effect of selected solvents (with dielectric constants, ɛ, from 1 to 109, Table 1) on various properties of aminopurines.

The amino group was characterized by both geometric (bond lengths and angles) and electronic parameters. As for the geometric parameters (Scheme 3), lengths of bonds between the NH_2_ and *ipso* atom of purine (C or N) (*d*_ipso-NH2_), and NH bonds in amino group (*d_NH_*) and pyramidalization (φ) of the NH_2_ group were investigated.

Electronic properties of the amino group were quantitatively described by the cSAR (charge of the substituent active region) parameter [50,51]. The cSAR(X) of the amino group was calculated as the sum of the charges at all atoms of the NH_2_ group and the charge at the ipso carbon or nitrogen atom, Equation (1).

(1) $$\mathrm{cSAR}\left({\mathrm{NH}}_{2}\right)=q\left({\mathrm{NH}}_{2}\right)+q{\left(C\;\mathrm{or}\;N\right)}_{\mathrm{ipso}}$$

The values of cSAR are positive for electron-donating groups; the higher the values, the more electron-donating the substituent is in a given system. Oppositely, for electron-withdrawing groups, cSAR has negative values, which are more negative with an increase of electron-withdrawing ability. Hirshfeld atomic charges were used in the calculation of cSAR [70]. 

Changes in π-electron delocalization in both purine rings were assessed using the geometry-based HOMA (Harmonic Oscillator Model of Aromaticity) index [71]. It is defined in Equation (2):

(2) $$\mathrm{HOMA}=1-\frac{1}{n}\sum_{i}^{n}\alpha_{j}{\left(d_{\mathrm{opt},j}-d_{j,i}\right)}^{2},$$

where *n* is the number of bonds taken into account when carrying out the summation, *i* means the type of bond (CC or CN), *α*_j_ is an empirical normalization constant (for CC and CN bonds, *α*_CC_ = 257.7 and *α*_CN_ = 93.52 from Ref. [72]), *d*_opt_,_j_ is the optimal length of a given bond assumed to be realized in fully aromatic systems with HOMA = 1 (for CC and CN bonds, *d*_opt,CC_ = 1.388 Å and *d*_opt,CN_ = 1.334 Å), and *d*_j,i_ is an actual bond length in the studied system. The NCI (non-covalent interaction index) method was used to detect possible noncovalent intramolecular interactions [73]; their nature was determined on the basis of the value of sgn(λ_2_)·*ρ*(r), where λ_2_ is the second eigenvalue of the Hessian matrix of electron density (*ρ*(r)). NCI analyses were performed in Multiwfn [74] and visualization in VMD program [75].

## 4. Conclusions

The aim of the work was to investigate changes in the properties of the amino group attached to the C2, C6, C8, or N (N1, N3, N7, or N9) position in the four most stable purine tautomers in various solvents, as well as the effect of substitution on the tautomer stability and π-electron structure of the purine rings. It was shown that, due to the position of substitution, tautomeric form, and solvent effect, changes in the electron-donating power of the NH_2_ group are significant; its cSAR (charge of the substituent active region) value ranges from 0.133 to 0.375. Thus, for substituted purine derivatives, the reverse substituent effect of the amino group is much greater than previously found for the electron-accepting nitro group [59]. 

It was found that the solvent effect’s strength depends on the proximity interactions of the amino group present in the given tautomeric form of aminopurine. In systems with repulsive proximity interactions (NH∙∙∙HN), a much stronger solvent effect is found than for derivatives with two attractive interactions (NH∙∙∙N). This is evidenced by appropriate changes in the electronic and geometric parameters of the amino group.

Substitution and solvation also affect π-electron delocalization in aminopurine rings, and the effect varies depending on the substitution position and tautomer; larger changes in aromaticity occur in the N1H and N3H tautomers than in N7H and N9H. In most cases, the aromatic character increases with the solvent polarity. Substitution with the amino group has a distinctly opposite effect on aromaticity than with the nitro group.

In 2-, 6-, and N-aminopurines, an increase in solvent polarity significantly reduces the differences in stability between the most and least stable tautomers; however, the tautomeric preferences do not change compared to unsubstituted purine. In 8-aminopurines, the difference in water is only 4 kcal/mol; additionally, the sequence of stability changes from N9H > N7H > N3H > N1H (gas phase) to N7H > N9H > N1H > N3H (water).

## Figures and Tables

**Table 1:** Values of dielectric constants (ε) of studied solvents.

Solvent/Medium	Acronym	ε	1/ε
Formamide	FA	108.94	0.0092
Water	H_2_O	78.36	0.0128
Dimethyl sulfoxide	DMSO	46.83	0.0214
Ethanol	EtOH	24.85	0.0402
Pyridine	Py	12.98	0.0771
Tetrahydrofuran	THF	7.43	0.1347
o-cresol	o-Cr	6.76	0.1479
Chloroform	Chf	4.71	0.2123
Toluene	Tol	2.37	0.4212
Gas phase	GP	1.00	1.00

**Table 2:** Statistical data on cSAR(NH_2_) = *a*∙(1/ε) + b linear fitting equations for C2-, C6-, C8-, and N-NH_2_-substituted 9H, 7H, 3H, and 1H purine tautomers; Δ is the difference between the value of cSAR(NH_2_) in H_2_O and the gas phase. Mixed-proximity systems in bold.

		Δ_cSAR(NH2)_	*a*	*R* ^2^	Proximity Type
C2	9H	0.0102	−0.0104	0.9964	2× att
	7H	−0.0007	0.0008	0.0585	2× att
	**3H**	**0.0956**	**−0.0979**	**0.9744**	**mix**
	**1H**	**0.0919**	**−0.0947**	**0.9691**	**mix**
C6	9H	0.0238	−0.0244	0.9989	2× att
	**7H**	**0.0795**	**−0.0818**	**0.9415**	**mix**
	3H	0.0359	−0.0367	0.9877	2× att
	**1H**	**0.1163**	**−0.1189**	**0.9668**	**mix**
C8	**9H**	**0.0747**	**−0.0766**	**0.9703**	**mix**
	**7H**	**0.0823**	**−0.0846**	**0.9692**	**mix**
	3H	0.0132	−0.0134	0.9947	2× att
	1H	−0.0032	0.0035	0.3025	2× att
N	**N9**	**0.0449**	**−0.0460**	**0.9780**	**mix**
	N7	0.0623	−0.0638	0.9725	2× rep
	**N3**	**0.0510**	**−0.0523**	**0.9795**	**mix**
	N1	0.0789	−0.0808	0.9121	2× rep

**Table 3:** Tautomer energies (in kcal/mol), relative to the 9H (*E*_rel_), for all considered substitution positions of purines (X = NH_2_, H, and NO_2_) in the gas (GP) and aqueous (H_2_O) phases. Values of ∆ indicate the change due to solvation, ∆ = *E*_rel_(H_2_O)–*E*_rel_(GP).

		X = NH_2_			X = NO_2_ *		
		GP	H_2_O	∆	GP	H_2_O	∆
C2	9H	0.00	0.00	0.00	0.00	0.00	0.00
	7H	3.59	0.71	−2.88	3.33	−0.31	−3.64
	3H	12.25	7.80	−4.45	8.17	11.60	3.43
	1H	15.92	7.47	−8.45	9.69	9.36	−0.33
C6	9H	0.00	0.00	0.00	0.00	0.00	0.00
	7H	7.18	1.91	−5.27	−4.06	−2.12	1.94
	3H	7.09	4.74	−2.35	10.16	7.99	−2.17
	1H	16.94	7.69	−9.25	10.34	9.81	−0.53
C8	9H	0.00	0.00	0.00	0.00	0.00	0.00
	7H	2.62	−0.69	−3.31	3.67	0.90	−2.77
	3H	2.95	3.50	0.55	8.96	2.10	−6.86
	1H	4.93	1.80	−3.13	11.55	−0.08	−11.63
N	N9	0.00	0.00	0.00			
	N7	4.98	0.90	−4.07			
	N3	7.11	5.86	−1.25			
	N1	13.03	6.14	−6.89			
PU	9H	0.00 **	0.00	0.00			
	7H	3.29 **	0.15	−3.14			
	3H	8.69 **	6.70	−1.99			
	1H	11.50 **	4.98	−6.52			

* Data taken from [59]. ** Data taken from [65].

**Table 4:** Statistical data (*a* and *R*^2^) of *E*_solv_ = *a*∙(1/ε) + *b* linear fitting equations, where 1/ε is the reciprocal of solvent dielectric constant (Table 1). Dipole moments for geometry in the gas phase, *μ*, and their changes due to solvation, ∆*μ* = *μ*(FA) – *E*(GP). All data concern NH_2_ derivatives of purine. C-NH_2_ systems with mixed proximity type are marked in bold.

		*a*	*R* ^2^	*μ/D*	Δ*μ/D*	Proximity Type
9H	C2	8.905	0.979	3.2	1.3	2× att
	C6	8.949	0.981	2.4	1.0	2× att
	C8	**11.211**	**0.976**	**5.4**	**2.8**	**mix**
	N9	7.585	0.982	3.1	1.2	mix
7H	C2	11.869	0.970	4.1	2.0	2× att
	C6	**14.364**	**0.964**	**6.8**	**3.6**	**mix**
	C8	**14.611**	**0.968**	**6.9**	**3.6**	**mix**
	N7	11.765	0.973	6.2	2.4	2× rep
3H	C2	**13.466**	**0.976**	**6.0**	**3.3**	**mix**
	C6	11.353	0.981	3.9	1.8	2× att
	C8	10.645	0.978	3.0	1.3	2× att
	N3	8.855	0.977	3.1	2.4	mix
1H	C2	**17.581**	**0.969**	**7.7**	**4.5**	**mix**
	C6	**18.453**	**0.967**	**8.2**	**4.8**	**mix**
	C8	14.432	0.972	4.1	2.4	2× att
	N1	14.650	0.974	7.0	3.1	2× rep

## Data Availability

All data generated in this study are presented in the Manuscript and its Supplementary Materials.

## Supplementary Materials

The following supporting information can be downloaded at https://www.mdpi.com/article/10.3390/molecules28072993/s1, Table S1: Energy values and the difference between the coplanar and perpendicular conformations of NH_2_ in N-NH_2_ derivatives of purine; Table S2: The cSAR(NH_2_) values for C2-, C6-, C8-, and N-NH_2_-substituted purine tautomers; Table S3: The valence angles at the N atom and pyramidalization of the NH_2_ group in the gas phase and water, their difference, and type of proximity for C2-, C6-, C8-, and N-NH_2_-substituted purine tautomers; Table S4: dipso_-NH2_ bond lengths in the gas phase and water, their difference, and type of proximity for C2-, C6-, C8-, and N-NH_2_-substituted purine tautomers; Table S5: *d_NH_* bond lengths in the gas phase and formamide, their difference, and type of proximity for C2-, C6-, C8-, and N-NH_2_-substituted purine tautomers; Table S6: Distance between H atom from NH_2_ and N atom from the purine ring, H···N, in the gas phase and formamide, their difference, and type of proximity for C2-, C6-, C8-, and N-NH_2_-substituted purine tautomers; Table S7: HOMA values of five- and six-membered rings in gas phase and formamide, their difference, and ∆HOMA_NH2PU-PU_ for purine and analyzed NH_2_-substituted derivatives of purine tautomers; Table S8: HOMA values of five- and six-membered rings (5MR, 6MR) in gas phase and formamide, their difference, and ∆HOMA_NO2PU-PU_ for analyzed NO_2_-substituted derivatives of purine tautomers; Table S9: Relative energies of C2-, C6-, C8-, and N-NH_2_-substituted purines; Table S10: Solvation energies of C2-, C6-, C8-, and N-NH_2_ coplanar-substituted purines; Table S11: Dipole moments, μ, of C2-, C6-, C8-, and N-NH_2_-substituted purines. Figure S1: NCI analysis for selected systems. Visualization of reduced density gradient isosurfaces; Figure S2: Change in (a) HOMA and (b) HOMED of 6- and 5-membered rings between the formamide and the gas phase plotted against the HOMA (HOMED) value for the respective ring of aminopurines in the gas phase; Figure S3: Values of HOMED index for 6- and 5-membered rings of C2, C6, C8, and N amino-substituted derivatives of purine in four tautomeric forms, calculated for the gas phase geometry; Figure S4: Changes in HOMED index due to (a) solvation and (b) substitution; Figure S5: Solvation energies of tautomers, *E*_solv_, for (a) C2-NH_2_, (b) C6-NH_2_ (c) C8-NH_2_ (d) N-NH_2_, and (e) unsubstituted purines.

## Sample Availability

All calculation files are available from the authors.

## References

[1] Rosemeyer H. (2004). The Chemodiversity of Purine as a Constituent of Natural Products. Chem. Biodivers..

[2] Covarrubias A.J., Perrone R., Grozio A., Verdin E. (2021). NAD+ metabolism and its roles in cellular processes during ageing. Nat. Rev. Mol. Cell Biol..

[3] Legraverend M., Grierson D.S. (2006). The purines: Potent and versatile small molecule inhibitors and modulators of key biological targets. Bioorganic Med. Chem..

[4] Pozharskiĭ A.F., Soldatenkov A.T., Katritzky A.R. (2011). Heterocycles in Life and Society: An Introduction to Heterocyclic Chemistry, Biochemistry, and Applications.

[5] Raczyńska E.D., Kamińska B. (2013). Variations of the tautomeric preferences and π-electron delocalization for the neutral and redox forms of purine when proceeding from the gas phase (DFT) to water (PCM). J. Mol. Model..

[6] Raczyńska E.D., Kamińska B. (2010). Prototropy and π-electron delocalization for purine and its radical ions—DFT studies. J. Phys. Org. Chem..

[7] Peral F., Gallego E. (2000). A study by ultraviolet spectroscopy on self-association of purine, 6-methylpurine, benzimidazole, and imidazo [1,2-a]pyridine in aqueous solution. Spectrochim. Acta.

[8] Gonnella N.C., Roberts J.D. (1982). Studies of the tautomerism of purine and the protonation of purine, and its 7- and 9-methyl derivatives, by nitrogen-15 nuclear magnetic resonance spectroscopy. J. Am. Chem. Soc..

[9] Burova T.G., Ten G.N., Kucherova V.V. (2004). Investigations of Tautomeric Purine Forms by the Methods of Vibrational Spectroscopy and Resonance Raman Scattering. II. Quantum-Mechanical Calculations of the Resonance Raman Scattering Spectra of Purine Tautomers. Russ. Phys. J..

[10] Nowak M.J., Lapinski L., Kwiatkowski J.S. (1989). An infrared matrix isolation study of tautomerism in purine and adenine. Chem. Phys. Lett..

[11] Nowak M.J., Rostkowska H., Lapinski L., Kwiatkowski J.S., Leszczynski J. (1994). Tautomerism N(9)H .dblharw. N(7)H of Purine, Adenine, and 2-Chloroadenine: Combined Experimental IR Matrix Isolation and Ab Initio Quantum Mechanical Studies. J. Phys. Chem..

[12] Chenon M.T., Pugmire R.J., Grant D.M., Panzica R.P., Townsend L.B. (1975). Carbon-13 magnetic resonance. XXVI. Quantitative determination of the tautomeric populations of certain purines. J. Am. Chem. Soc..

[13] Sečkářová P., Marek R., Maliňáková K., Kolehmainen E., Hocková D., Hocek M., Sklenář V. (2004). Direct determination of tautomerism in purine derivatives by low-temperature NMR spectroscopy. Tetrahedron Lett..

[14] Lippert B., Gupta D. (2009). Promotion of rare nucleobase tautomers by metal binding. Dalton Trans..

[15] Raczyńska E.D., Gal J.-F., Maria P.-C., Kamińska B., Igielska M., Kurpiewski J., Juras W. (2020). Purine tautomeric preferences and bond-length alternation in relation with protonation-deprotonation and alkali metal cationization. J. Mol. Model..

[16] Ouellette R.J., Rawn J.D. (2018). Nucleosides, Nucleotides, and Nucleic Acids. Organic Chemistry: Structure, Mechanism, Syn-Thesis.

[17] Yang B., Srinivasan B. (2014). Recent Developments in the Synthesis of Substituted Purine Nucleosides and Nucleotides. Curr. Org. Chem..

[18] Ratsep P.C., Robins R.K., Vaghefi M.M. (1990). Introduction of Fluorine Into the C8 Position of Purine Nucleosides. Nucleosides Nucleotides.

[19] Galmarini C.M., Mackey J.R., Dumontet C. (2002). Nucleoside analogues and nucleobases in cancer treatment. Lancet Oncol..

[20] Walters W.P., Murcko M.A., Böhm H., Schneider G. (2000). Library Filtering Systems and Prediction of Drug-Like Properties. Methods and Principles in Medicinal Chemistry.

[21] Naumann T., Matter H. (2002). Structural Classification of Protein Kinases Using 3D Molecular Interaction Field Analysis of Their Ligand Binding Sites: Target Family Landscapes. J. Med. Chem..

[22] Schulze-Gahmen U., Brandsen J., Jones H.D., Morgan D., Meijer L., Vesely J., Kim S.-H. (1995). Multiple modes of ligand recognition: Crystal structures of cyclin-dependent protein kinase 2 in complex with ATP and two inhibitors, olomoucine and isopentenyladenine. Proteins: Struct. Funct. Genet..

[23] Donohue J., Trueblood K.N. (1960). Base pairing in deoxyribonucleic acid. J. Mol. Biol..

[24] Freese E. (1959). The specific mutagenic effect of base analogues on Phage T4. J. Mol. Biol..

[25] Drake J.W. (1970). The Molecular Basis of Mutation.

[26] Ludwig V., Amaral M.S.D., da Costa Z.M., Borin A.C., Canuto S., Serrano-Andrés L. (2008). 2-Aminopurine non-radiative decay and emission in aqueous solution: A theoretical study. Chem. Phys. Lett..

[27] Glickman B.W., de Serres F.J. (1985). 2-Aminopurine Mutagenesis in *Escherichia coli*. Genetic Consequences of Nucleotide Pool Imbalance, Basic Life Sciences.

[28] Ronen A. (1980). 2-Aminopurine. Mutat. Res./Rev. Mutat. Res..

[29] de Serres F.J., Brockman H.E., Hung C.Y., Overton L.K., de Serres F.J. (1985). Mutagenicity of 2-aminopurine, 6-N-hydroxylaminopurine, and 2-amino-N6-hydroxyadenine in *Neurospora crassa*. Genetic Consequences of Nucleotide Pool Imbalance, Basic Life Sciences.

[30] Laufer S.A., Domeyer D.M., Scior T.R.F., Albrecht W., Hauser D.R.J. (2005). Synthesis and Biological Testing of Purine Derivatives as Potential ATP-Competitive Kinase Inhibitors. J. Med. Chem..

[31] Baker B., Hendrickson J. (1967). Irreversible enzyme inhibitors XCII. Inhibition of xanthine oxidase by some purines and pyr midines. J. Pharm. Sci..

[32] Vanotti E., Bani M., Favara D., Gobetti M., Lombroso M., Magnetti S., Olgiati V., Palladino M., Tonon G. (1994). 8-Substituted purine derivatives: A new class of lipid-lowering agents. Eur. J. Med. Chem..

[33] Huryn D.M., Okabe M. (1992). AIDS-driven nucleoside chemistry. Chem. Rev..

[34] Seela F., Winter H. (1994). N7-DNA: Synthesis and Base Pairing of Oligonucleotides containing 7-(2-Deoxy-D-erythro-pentofuranosyl)adenine (N7Ad). Helv. Chim. Acta.

[35] Boryski J. (1996). Transglycosylation Reactions of Purine Nucleosides. A Review. Nucleosides Nucleotides.

[36] Marek R., Brus J., Toušek J., Kovács L., Hocková D. (2002). N^7^- and N^9^-substituted purine derivatives: A ^15^n NMR study. Org. Magn. Reson..

[37] Arico J.W. (2010). 3-Substituted Purines: Methodology, Synthesis, and Studies of DNA Hydration in the Minor Groove. Ph.D. Thesis.

[38] Raczyńska E.D., Makowski M. (2014). Geometric consequences of electron delocalization for adenine tautomers in aqueous solution. J. Mol. Model..

[39] Mburu E., Matsika S. (2008). An Ab Initio Study of Substituent Effects on the Excited States of Purine Derivatives. J. Phys. Chem. A.

[40] Palamarchuk G.V., Shishkin O.V., Gorb L., Leszczynski J. (2013). Nucleic Acid Bases in Anionic 2′-Deoxyribonucleotides: A DFT/B3LYP Study of Structures, Relative Stability, and Proton Affinities. J. Phys. Chem. B.

[41] Tomasi J., Mennucci B., Cammi R. (2005). Quantum Mechanical Continuum Solvation Models. Chem. Rev..

[42] Miertuš S., Scrocco E., Tomasi J. (1981). Electrostatic interaction of a solute with a continuum. A direct utilizaion of AB initio molecular potentials for the prevision of solvent effects. Chem. Phys..

[43] Miertuš S., Tomasi J. (1982). Approximate evaluations of the electrostatic free energy and internal energy changes in solution processes. Chem. Phys..

[44] Hammett L.P. (1940). Physical Organic Chemistry, Table II.

[45] Hammett L.P. (1937). The Effect of Structure upon the Reactions of Organic Compounds. Benzene Derivatives. J. Am. Chem. Soc..

[46] Hansch C., Leo A., Taft R.W. (1991). A survey of Hammett substituent constants and resonance and field parameters. Chem. Rev..

[47] Exner O. (2006). Theory of Substituent Effects: Recent Advances. Curr. Org. Chem..

[48] Galabov B., Ilieva S., Schaefer I.H.F. (2006). An Efficient Computational Approach for the Evaluation of Substituent Constants. J. Org. Chem..

[49] Krygowski T.M., Zachara J.E., Szatylowicz H. (2004). Molecular Geometry as a Source of Chemical Information. 3. How H-Bonding Affects Aromaticity of the Ring in the Case of Phenol and *p*-Nitrophenol Complexes: A B3LYP/6-311+G** Study. J. Org. Chem..

[50] Sadlej-Sosnowska N. (2007). On the way to physical interpretation of Hammett constants: How substituent active space impacts on acidity and electron distribution in p-substituted benzoic acid molecules. Polish J. Chem..

[51] Sadlej-Sosnowska N. (2007). Substituent active region—A gate for communication of substituent charge with the rest of a molecule: Monosubstituted benzenes. Chem. Phys. Lett..

[52] Stasyuk O.A., Szatylowicz H., Guerra C.F., Krygowski T.M. (2015). Theoretical study of electron-attracting ability of the nitro group: Classical and reverse substituent effects. Struct. Chem..

[53] Szatylowicz H., Siodla T., Stasyuk O.A., Krygowski T.M. (2015). Towards physical interpretation of substituent effects: The case of meta- and para-substituted anilines. Phys. Chem. Chem. Phys..

[54] Szatyłowicz H., Krygowski T.M., Hobza P. (2006). How the Shape of the NH_2_ Group Depends on the Substituent Effect and H-Bond Formation in Derivatives of Aniline. J. Phys. Chem. A.

[55] Szatylowicz H., Jezuita A., Siodła T., Varaksin K.S., Ejsmont K., Madura I.D., Krygowski T.M. (2018). Dependence of the Substituent Effect on Solvent Properties. J. Phys. Chem. A.

[56] Iii G.L.D., Je L., Tanski J.M. (2019). Crystallographic and spectroscopic characterization of 4-nitro-2-(trifluoromethyl)benzoic acid and 4-nitro-3-(trifluoromethyl)benzoic acid. Acta Crystallogr. Sect. E Crystallogr. Commun..

[57] Hanson J.R., Hitchcock P.B., Toche F. (2008). The Rotation of the Nitro and Formyl Groups Relative to the Aromatic Ring in Some ortho-nitroarylaldehydes. J. Chem. Res..

[58] Dobrowolski M.A., Krygowski T.M., Cyrański M.K. (2009). Substituent Constants (σp−) of the Rotated Nitro Group. The In-terplay Between the Substituent Effect of a Rotated−NO_2_ Group and H-Bonds Affecting π-Electron Delocalization in 4-Nitrophenol and 4-Nitrophenolate Complexes: A B3LYP/6-311+ G** Study. Croat. Chem. Acta.

[59] Jezuita A., Wieczorkiewicz P., Szatylowicz H., Krygowski T. (2021). Solvent Effect on the Stability and Reverse Substituent Effect in Nitropurine Tautomers. Symmetry.

[60] Jezuita A., Wieczorkiewicz P.A., Szatylowicz H., Krygowski T.M. (2021). Effect of the Solvent and Substituent on Tautomeric Preferences of Amine-Adenine Tautomers. ACS Omega.

[61] Wieczorkiewicz P.A. (2022). Relationships between the Substituent and Solvent Effects in Selected Pyrimidine and Purine Derivatives. Master’s Thesis.

[62] Desiraju G.R. (2010). A Bond by Any Other Name. Angew. Chem. Int. Ed..

[63] Raczyńska E.D., Hallman M., Kolczynska K., Stępniewski T.M. (2010). On the Harmonic Oscillator Model of Electron Delocalization (HOMED) Index and its Application to Heteroatomic π-Electron Systems. Symmetry.

[64] Szczȩśniak M., Nowak M., Szczepaniak K. (1984). Infrared matrix isolation studies on tautomerism of cytosine and isocytosine methyl-derivatives. J. Mol. Struct..

[65] Jezuita A., Szatylowicz H., Marek P.H., Krygowski T.M. (2019). Aromaticity of the most stable adenine and purine tautomers in terms of Hückel’s 4n+2 principle. Tetrahedron.

[66] Frisch M.J., Trucks G.W., Schlegel H.B., Scuseria G.E., Robb M.A., Cheeseman J.R., Scalmani G., Barone V., Mennucci B., Petersson G.A. (2013). Gaussian 09, Revision D.01.

[67] Grimme S., Antony J., Schwabe T., Mück-Lichtenfeld C. (2007). Density functional theory with dispersion corrections for supramolecular structures, aggregates, and complexes of (bio)organic molecules. Org. Biomol. Chem..

[68] Dunning T.H. (1989). Gaussian basis sets for use in correlated molecular calculations. I. The atoms boron through neon and hydrogen. J. Chem. Phys..

[69] Marek P.H., Szatylowicz H., Krygowski T.M. (2018). Stacking of nucleic acid bases: Optimization of the computational approach—the case of adenine dimers. Struct. Chem..

[70] Hirshfeld F.L. (1977). Bonded-atom fragments for describing molecular charge densities. Theor. Chim. Acta.

[71] Kruszewski J., Krygowski T. (1972). Definition of aromaticity basing on the harmonic oscillator model. Tetrahedron Lett..

[72] Krygowski T.M. (1993). Crystallographic studies of inter- and intramolecular interactions reflected in aromatic character of .pi.-electron systems. J. Chem. Inf. Comput. Sci..

[73] Johnson E.R., Keinan S., Mori-Sánchez P., Contreras-García J., Cohen A.J., Yang W. (2010). Revealing Noncovalent Interactions. J. Am. Chem. Soc..

[74] Lu T., Chen F. (2012). Multiwfn: A multifunctional wavefunction analyzer. J. Comput. Chem..

[75] Humphrey W., Dalke A., Schulten K. (1996). VMD: Visual molecular dynamics. J. Mol. Graph..

