# Supplementary material for: Influence of the Solvent on the Stability of Aminopurine Tautomers and Properties of the Amino Group
Source: Molecules. 2023 Mar 27;28(7):2993. doi: 10.3390/molecules28072993 (PMC10095612; doi:10.3390/molecules28072993)
Supplement: Supplementary file 1 [file molecules-28-02993-s001.zip › aminoPU-molecules-SM-27-03-2023.pdf]

Supplementary Materials (SM)

Influence of the solvent on the stability of aminopurine tautomers and properties of the amino group

Anna Jezuita[1], Paweł A. Wieczorkiewicz[2,*], Tadeusz M. Krygowski[3] and Halina Szatylowicz[2,*]

[1] Faculty of Science and Technology, Jan Dlugosz University in Czestochowa, Al. Armii Krajowej 13/15, 42-200 Czestochowa, Poland
[2] Faculty of Chemistry, Warsaw University of Technology, Noakowskiego 3, 00-664 Warsaw, Poland
[3] Department of Chemistry, Warsaw University, Pasteura 1, 02-093 Warsaw, Poland
* Correspondence: pawel.wieczorkiewicz.dokt@pw.edu.pl (P.A.W.); halina.szatylowicz@pw.edu.pl (H.S.)

**Table S1.** Energy values and the difference, Δ, between the coplanar and perpendicular conformations of NH_2 in N-NH_2 derivatives of purine ……………………….	**SM2**
**Table S2.** The cSAR(NH_2) values for C2-, C6-, C8- and NX-NH_2 substituted purine tautomers	SM3
**Table S3.** The valence angles at the N atom and pyramidalization, angle (φ), values of the NH_2 group (°) in the gas phase (GP) and water (H_2O), their difference, Δ, and type of proximity for C2-, C6-, C8- and NX-NH_2 substituted purine tautomers …………………………………..	SM4
**Table S4.** $d_{ipso\text{-}NH2}$ bond lengths in the gas phase (GP) and water (H_2O), their difference, Δ, and type of proximity for C2-, C6-, C8- and NX-NH_2 substituted purine tautomers …………	SM4
**Table S5.** d_{NH} bond lengths (in Å) in the gas phase (GP) and formamide (FA), their difference, Δ, and type of proximity for C2-, C6-, C8- and N-NH_2 substituted purine tautomers…………	SM5
**Table S6.** Distance between H atom from NH_2 and N atom from the purine ring, H···N, in the gas phase (GP) and formamide (FA), their difference, Δ, and type of proximity for C2-, C6-, C8- and N-NH_2 substituted purine tautomers ……………….……………………………...	SM6
**Table S7**. HOMA values of five- and six-membered rings in gas phase (GP) and formamide (FA), their difference, Δ, and $\Delta HOMA_{NH2PU\text{-}PU}$, for purine and analyzed NH_2-substituted derivatives of purine tautomers……………………………………………….……………..	SM7
**Table S8**. HOMA values of five- and six-membered rings in gas phase (GP) and formamide (FA), their difference, Δ, and $\Delta HOMA_{NO2PU\text{-}PU}$ for analyzed NO_2-substituted derivatives of purine tautomers……………………………………………………………………………	SM8
**Table S9**. Relative energies of C2-, C6-, C8- and N-NH_2 substituted purines………………..	SM9
**Table S10**. Solvation energies of C2-, C6-, C8- and N-NH_2 coplanar substituted purines..........	SM10
**Table S11**. Dipole moments, μ, of C2-, C6-, C8- and N-NH_2 substituted purines……………..	SM11
**Figure S1**. NCI analysis for selected systems. Visualization of reduced density gradient isosurfaces.…………………………………………………….………………………	SM12
**Figure S2**. Change in (a) HOMA and (b) HOMED of 6- and 5-membered rings between the most polar solvent, formamide (FA) and the gas phase plotted against the HOMA value for the respective ring of aminopurines in the gas phase……………………………………………..	SM13
**Calculation of HOMED index in NH_2-substituted purine**…..…………………………...	SM14
**Figure S3**. Values of HOMED index for 6- and 5-membered rings of C2, C6, C8 and N-amino-substituted derivatives of purine in four tautomeric forms, for the gas phase geometry	SM14
**Figure S4**. Changes in HOMED index due to (a) solvation and (b) substitution……………...	SM15
**Figure S5.** Solvation energies of tautomers, E_{solv}, for (a) C2-NH_2, (b) C6-NH_2 (c) C8-NH_2 (d) N-NH_2 and (e) unsubstituted purines………………………………………………………..	SM16

Table S1. Energy values and the difference, Δ, in free energy values (in kcal/mol) between the coplanar (X||) and perpendicular (X⊥) conformations of NH_2 relative to the plane of purine ring in N-NH_2 derivatives of purine.

	GP		H_2O		GP	H_2O
	N-X||	N-X⊥	N-X||	N-X⊥	Δ*	
1H	-293055.94	-293058.74	-293071.50	-293073.05	-2.80	-1.55
3H	-293055.68	-293064.67	-293073.01	-293073.33	-8.99	-0.32
7H	-293063.05	-293066.80	-293075.38	-293078.28	-3.75	-2.91
9H	-293068.16	-293071.78	-293076.60	-293079.19	-3.62	-2.59

*Δ = N-X⊥ – N-X||

Table S2. The cSAR(NH_2) values for C2-, C6-, C8- and N-NH_2 substituted purine tautomers.

		GP	Tol	Chf	o-Cr	THF	Py	EtOH	DMSO	H_2O	FA	Δ	*average*
9H	**C2**	0.2514	0.2576	0.2600	0.2606	0.2607	0.2612	0.2614	0.2615	0.2616	0.2616	*0.0102*	*0.2597*
7H		0.2433	0.2453	0.2449	0.2444	0.2443	0.2436	0.2431	0.2428	0.2426	0.2425	*0.0028*	*0.2437*
3H		0.2784	0.3212	0.3445	0.3530	0.3548	0.3633	0.3692	0.3725	0.3740	0.3746	*0.0963*	*0.3506*
1H		0.2524	0.2914	0.3155	0.3241	0.3259	0.3342	0.3399	0.3429	0.3443	0.3449	*0.0925*	*0.3215*
9H	**C6**	0.2405	0.2537	0.2594	0.2611	0.2614	0.2629	0.2637	0.2642	0.2643	0.2644	*0.0239*	*0.2596*
7H		0.1841	0.2145	0.2339	0.2422	0.2441	0.2532	0.2592	0.2622	0.2636	0.2642	*0.0801*	*0.2421*
3H		0.2696	0.2870	0.2957	0.2986	0.2993	0.3021	0.3040	0.3050	0.3054	0.3056	*0.0361*	*0.2972*
1H		0.2439	0.2941	0.3225	0.3328	0.3351	0.3458	0.3536	0.3581	0.3602	0.3612	*0.1173*	*0.3307*
9H	**C8**	0.2595	0.2920	0.3105	0.3174	0.3189	0.3258	0.3305	0.3330	0.3342	0.3347	*0.0751*	*0.3157*
7H		0.2702	0.3057	0.3262	0.3340	0.3357	0.3433	0.3485	0.3512	0.3524	0.3530	*0.0828*	*0.3320*
3H		0.2734	0.2817	0.2847	0.2855	0.2856	0.2862	0.2865	0.2866	0.2866	0.2867	*0.0132*	*0.2843*
1H		0.2723	0.2745	0.2732	0.2723	0.2721	0.2709	0.2699	0.2693	0.2690	0.2689	*0.0056*	*0.2712*
9H	**N-X||**	0.1327	0.1533	0.1642	0.1682	0.1690	0.1729	0.1756	0.1770	0.1777	0.1779	*0.0452*	*0.1668*
7H		0.1389	0.1664	0.1816	0.1873	0.1885	0.1941	0.1980	0.2001	0.2011	0.2015	*0.0627*	*0.1858*
3H		0.2021	0.2256	0.2380	0.2425	0.2434	0.2478	0.2508	0.2524	0.2531	0.2534	*0.0513*	*0.2409*
1H		0.1792	0.2142	0.2335	0.2406	0.2422	0.2493	0.2542	0.2568	0.2581	0.2586	*0.0794*	*0.2387*
9H	**N-X⊥**	0.1272	0.1448	0.1539	0.1572	0.1579	0.1611	0.1633	0.1645	0.1650	0.1652	*0.0381*	*0.1560*
7H		0.1303	0.1700	0.1709	0.1761	0.1772	0.1823	0.1859	0.1877	0.1886	0.1890	*0.0587*	*0.1758*
3H		0.1749	0.1944	0.2081	0.2150	0.2161	0.2215	0.2282	0.2310	0.2321	0.2326	*0.0576*	*0.2154*
1H		0.1673	0.1981	0.2144	0.2204	0.2217	0.2276	0.2317	0.2338	0.2349	0.2353	*0.0680*	*0.2185*
range		*0.264*	*0.293*	*0.311*	*0.317*	*0.318*	*0.323*	*0.327*	*0.329*	*0.330*	*0.330*		
range	**C2**	0.0351	0.0759	0.0996	0.1085	0.1105	0.1197	0.1262	0.1297	0.1314	0.1321	*min*	0.1327*
	**C6**	0.0854	0.0796	0.0885	0.0906	0.0910	0.0926	0.0944	0.0959	0.0966	0.0970	*max*	0.3746
	**C8**	0.0139	0.0312	0.0529	0.0616	0.0635	0.0724	0.0786	0.0819	0.0834	0.0841	*Δ*	0.2419*
	**N-X||**	0.0693	0.0724	0.0738	0.0743	0.0744	0.0764	0.0786	0.0798	0.0804	0.0807	*without N-X⊥	
	**N-X⊥**	0.0478	0.0533	0.0605	0.0632	0.0638	0.0665	0.0684	0.0694	0.0699	0.0701		

Table S3. Valence angles at the N atom and pyramidalization, angle φ, values of the NH_2 group (°) in the gas phase (GP) and water (H_2O), their difference, Δ, and type of proximity for C2-, C6-, C8- and N-NH_2 substituted purine tautomers (see Scheme 2).

		GP				H_2O				Δ* φ	type of proximity
		$C_{ipso}NH_1$	$C_{ipso}NH_2$	H_1NH_2	φ	$C_{ipso}NH_1$	$C_{ipso}NH_2$	H_1NH_2	φ		
9H	**C2**	116.43	117.06	117.90	8.62	116.52	116.85	116.31	10.32	1.70	2x att
7H		116.29	116.43	117.59	9.68	116.00	116.11	115.64	12.25	2.57	2x att
3H		112.27	117.42	113.66	16.65	116.29	120.66	116.78	6.27	-10.38	mix
1H		110.53	115.76	111.66	22.05	119.28	114.86	115.00	10.86	-11.19	mix
9H	**C6**	118.33	119.37	119.63	2.67	118.96	119.75	118.48	2.81	0.14	2x att
7H		119.42	119.84	120.74	0.00	120.14	120.34	119.52	0.00	0.00	mix
3H		117.02	113.07	113.77	16.15	116.81	120.13	116.34	6.73	-9.41	2x att
1H		112.48	118.02	113.79	15.72	118.17	122.45	118.05	1.34	-14.38	mix
9H	**C8**	116.21	112.13	112.86	18.79	115.43	118.41	115.01	11.16	-7.64	mix
7H		116.69	112.55	113.37	17.38	119.41	116.52	116.11	7.96	-9.42	mix
3H		117.84	117.52	118.39	6.26	118.08	118.15	117.18	6.59	0.33	2x att
1H		118.00	117.84	118.85	5.30	117.77	117.76	116.85	7.62	2.31	2x att
9H	**N-X||**	104.44	106.12	104.68	44.76	106.43	105.26	105.19	43.12	-1.64	mix
7H		106.05	105.83	104.46	43.66	106.35	106.33	104.92	42.40	-1.27	2x rep
3H		106.77	108.38	110.77	34.08	106.93	108.15	108.70	36.21	2.13	mix
1H		106.38	106.21	105.00	42.41	107.05	106.87	105.66	40.42	-1.99	2x rep

*Δ = H_2O – GP

Table S4. d_{ipso}-$_{NH2}$ bond lengths (in Å) in the gas phase (GP) and water (H_2O), their difference, Δ, and type of proximity for C2-, C6-, C8- and N-NH_2 substituted purine tautomers (see Scheme 2).

		GP	H_2O	Δ* d_{ipso}-$_{NH2}$	proximity type
		C_{ipso}-NH_2	C_{ipso}-NH_2		
9H	**C2**	1.3750	1.3744	-0.001	2x att
7H		1.3776	1.3791	0.002	2x att
3H		**1.3834**	**1.3597**	-0.024	mix
1H		**1.3931**	**1.3687**	-0.024	mix
9H	**C6**	1.3616	1.3574	-0.004	2x att
7H		1.3861	1.3652	-0.021	mix
3H		1.3542	1.3487	-0.005	2x att
1H		1.3752	1.3468	-0.028	mix
9H	**C8**	**1.3786**	**1.3610**	-0.018	mix
7H		**1.3756**	**1.3553**	-0.020	mix
3H		1.3604	1.3579	-0.002	2x att
1H		1.3591	1.3605	0.001	2x att
9H	**N-X||**	1.4207	1.4158	-0.005	mix
7H		1.4249	1.4188	-0.006	2x rep
3H		1.4101	1.4072	-0.003	mix
1H		1.4294	1.4209	-0.009	2x rep

* Δ = H_2O – GP

Table S5. d_{NH} bond lengths (in Å) in the gas phase (GP) and formamide (FA), their difference, Δ, and type of proximity for C2-, C6-, C8- and N-NH_2 substituted purine tautomers (see Scheme 2).

		GP		FA		Δ* d_{NH}		proximity type
		N-H_1	N-H_2	N-H_1	N-H_2	N-H_1	N-H_2	
9H	**C2**	1.0117	1.0117	1.0131	1.0128	0.0014	0.0012	2x att
7H		1.0122	1.0118	1.0136	1.0134	0.0014	0.0016	2x att
3H		**1.0150**	1.0151	1.0124	1.0125	-0.0026	-0.0025	mix
1H		1.0169	1.01724	1.0137	1.0137	-0.0032	-0.0035	mix
9H	**C6**	1.0113	1.0108	1.0121	1.0114	0.0009	0.0006	2x att
7H		**1.0163**	1.0143	1.0136	1.0119	-0.0027	-0.0024	mix
3H		1.0104	1.0110	1.0117	1.0111	0.0013	0.0001	2x att
1H		**1.0154**	1.0169	1.0118	1.0116	-0.0036	-0.0053	mix
9H	**C8**	**1.0159**	1.0155	**1.0140**	1.0137	-0.0019	-0.0018	mix
7H		**1.0155**	1.0150	1.0129	1.0127	-0.0026	-0.0023	mix
3H		1.0110	1.0113	1.0121	1.0123	0.0011	0.0009	2x att
1H		**1.0109**	1.0110	1.0123	1.0126	0.0014	0.0016	2x att
9H	**N-X||**	**1.0277**	1.0263	**1.0259**	1.0251	-0.0018	-0.0012	mix
7H		**1.0247**	1.0262	1.0238	1.0250	-0.0009	-0.0012	2x rep
3H		1.0239	1.0211	1.0236	1.0221	-0.0003	0.0010	mix
1H		**1.0254**	1.0245	1.0242	1.0236	-0.0012	-0.0009	2x rep

* Δ = FA – GP.

Table S6. Distance (in Å) between H atom from NH2 and N atom from the purine ring, H···N, in the gas phase (GP) and formamide (FA), their difference, Δ, and type of proximity for C2-, C6-, C8- and N-NH_2 substituted purine tautomers (see Scheme 2).

2x att | **2x rep** | **mix**

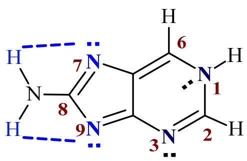

C8-NH_2 1H

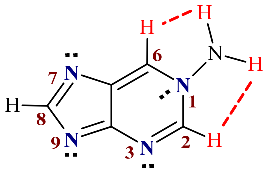

N1-NH_2 N1

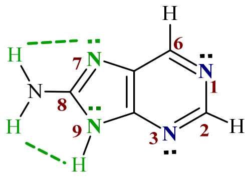

C8-NH_2 9H

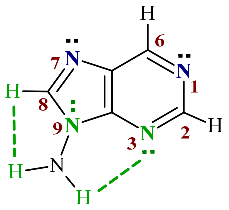

N9-NH_2 N9

		GP		FA		Δ*		proximity type
		H…$N_{7,3,1}$	H…$N_{9,1,7}$	H…$N_{7,3,1}$	H…$N_{9,7,3}$	H…$N_{7,3,1}$	H…$N_{9,1,7}$	
**C2**	9H	2.458	2.484	2.472	2.487	*0.013*	*0.003*	2x att
	7H	2.460	2.472	2.466	2.483	*0.006*	*0.011*	
**C6**	9H	2.515	2.817	2.525	2.832	*0.010*	*0.016*	
	3H	2.509	2.808	2.510	2.841	*0.001*	*0.033*	
**C8**	3H	2.579	2.560	2.594	2.569	*0.015*	*0.009*	
	1H	2.555	2.576	2.584	2.571	*0.029*	*0.005*	
		GP		FA		Δ*		
		H…HC_6	H…$HC_{8,2}$	H…HC_6	H…$HC_{8,2}$	H…HC_6	H…$HC_{8,2}$	
**N-X‖**	N7	2.543	2.657	2.554	2.619	*0.039*	*0.011*	2x rep
	N1	2.342	2.183	2.322	2.184	*0.001*	*0.020*	
		GP		FA		Δ*		
		H…$N_{9,7,3,1}$	H…$HN_{9,7,3,1}$	H…$N_{9,7,3,1}$	H…$HN_{9,7,3,1}$	H…$N_{9,7,3,1}$	H…$HN_{9,7,3,1}$	
**C2**	3H	2.436	2.436	2.482	2.369	*0.046*	*0.067*	mix
	1H	2.426	2.439	2.486	2.327	*0.060*	*0.113*	
**C6**	7H	2.444	2.750	2.491	2.682	*0.048*	*0.068*	
	1H	2.727	2.476	2.822	2.402	*0.096*	*0.074*	
**C8**	9H	2.538	2.673	2.589	2.588	*0.051*	*0.085*	
	7H	2.537	2.651	2.592	2.580	*0.055*	*0.071*	
		H…$N_{9,3}$	H… $HC_{8,2}$	H…$N_{9,3}$	H… $HC_{8,2}$	H…$N_{9,3}$	H… $HC_{8,2}$	
**N-X‖**	N9	2.597	2.737	2.646	2.669	*0.049*	*0.067*	
	N3	2.500	2.307	2.567	2.280	*0.067*	*0.027*	

* Δ = FA – GP

For example: NH_2 group interacts by H atoms as:
- in the case of C2-NH_2, 9H tautomer: H… N_3 and H…N_1
- in the case of N7-NH_2, N7 tautomer: H… HC_6 and H…HC_8
- in the case of C2-NH_2, 3H tautomer: H…N_1 and H…HN_3

Table S7. HOMA values of five- and six-membered rings (5_{MR}, 6_{MR}) in gas phase (GP) and formamide (FA), their difference, Δ, and $\Delta HOMA_{NH2PU-PU}$ obtained as difference between HOMA for substituted and unsubstituted purine systems in formamide (FA) and gas phase (GP), for analyzed NH_2-substituted derivatives of purine tautomers.

X=NH_2		HOMA						$\Delta HOMA_{NH2PU-PU}$			
		GP		FA		Δ**		GP		FA	
		6_{MR}	5_{MR}	6_{MR}	5_{MR}	6_{MR}	5_{MR}	6_{MR}	5_{MR}	6_{MR}	5_{MR}
9H		0.926*	0.757*	0.920	0.774	0.006	-0.017				
7H		0.915*	0.752*	0.917	0.793	-0.002	-0.041				
3H		0.761*	0.680*	0.812	0.726	-0.051	-0.046				
1H		0.602*	0.548*	0.719	0.744	-0.117	-0.196				
9H	**C2**	0.919	0.745	0.914	0.761	-0.005	0.017	-0.007	-0.012	-0.006	-0.013
7H		0.909	0.757	0.914	0.798	0.005	0.040	-0.006	0.005	-0.003	0.005
3H		0.801	0.708	0.856	0.766	0.055	0.057	0.040	0.028	0.044	0.040
1H		0.575	0.527	0.696	0.625	0.121	0.098	-0.027	-0.021	-0.022	-0.119
9H	**C6**	0.929	0.802	0.919	0.818	-0.010	0.016	0.003	0.045	-0.002	0.044
7H		0.927	0.776	0.919	0.822	-0.008	0.046	0.012	0.024	0.002	0.029
3H		0.850	0.840	0.862	0.850	0.012	0.010	0.089	0.160	0.051	0.124
1H		0.720	0.736	0.803	0.811	0.083	0.075	0.118	0.188	0.084	0.067
9H	**C8**	0.922	0.733	0.907	0.745	-0.015	0.013	-0.004	-0.024	-0.014	-0.029
7H		0.910	0.726	0.898	0.759	-0.012	0.034	-0.005	-0.026	-0.019	-0.034
3H		0.716	0.640	0.754	0.664	0.037	0.023	-0.045	-0.040	-0.058	-0.062
1H		0.569	0.493	0.676	0.582	0.107	0.088	-0.033	-0.055	-0.043	-0.163
9H	**N-X‖**	0.924	0.780	0.918	0.790	-0.006	0.010	-0.002	0.023	-0.002	0.015
7H		0.899	0.769	0.902	0.796	0.004	0.027	-0.016	0.017	-0.014	0.003
3H		0.813	0.736	0.843	0.766	0.030	0.030	0.052	0.056	0.031	0.040
1H		0.641	0.585	0.746	0.684	0.106	0.099	0.039	0.037	0.027	-0.061

* Data taken from Ref. [Jezuita, A.; Szatylowicz, H.; Marek, P.H.; Krygowski, T.M. Aromaticity of the Most Stable Adenine and Purine Tautomers in Terms of Hückel's 4n+2 Principle. *Tetrahedron* **2019**, *75(35)*, 130474; doi: 10.1016/j.tet.2019.130474]

** Δ = FA – GP

Table S8. HOMA values of five- and six-membered rings(5_{MR}, 6_{MR}) in gas phase (GP) and formamide (FA), their difference, Δ, and $\Delta HOMA_{NO2PU-PU}$ obtained as difference between HOMA for substituted and unsubstituted purine systems in formamide (FA) and gas phase (GP), for analyzed NO_2-substituted derivatives of purine tautomers with NO_2 group in-plane of purine ring.*

								$\Delta HOMA_{NO2PU-PU}$			
X=NO_2		GP		FA		Δ**		GP		FA	
		6_{MR}	5_{MR}	6_{MR}	5_{MR}	6_{MR}	5_{MR}	6_{MR}	5_{MR}	6_{MR}	5_{MR}
9H	C2	0.922	0.761	0.909	0.777	-0.013	0.016	-0.004	0.004	-0.011	0.003
7H		0.910	0.749	0.904	0.784	-0.006	0.035	-0.005	-0.003	-0.013	-0.009
3H		0.704	0.622	0.742	0.661	0.038	0.039	-0.057	-0.058	-0.070	-0.065
1H		0.600	0.530	0.697	0.624	0.097	0.094	-0.002	-0.018	-0.022	-0.120
9H	C6	0.876	0.724	0.861	0.735	-0.015	0.011	-0.050	-0.033	-0.059	-0.039
7H		0.882	0.735	0.874	0.747	-0.008	0.012	-0.033	-0.017	-0.043	-0.046
3H		0.661	0.560	0.703	0.605	0.042	0.045	-0.100	-0.120	-0.109	-0.121
1H		0.425	0.313	0.547	0.438	0.122	0.125	-0.177	-0.235	-0.172	-0.306
9H	C8	0.904	0.770	0.902	0.790	-0.002	0.02	-0.022	0.013	-0.018	0.016
7H		0.896	0.763	0.903	0.804	0.007	0.041	-0.019	0.011	-0.014	0.011
3H		0.782	0.730	0.833	0.780	0.051	0.05	0.021	0.050	0.021	0.054
1H		0.626	0.601	0.742	0.702	0.116	0.101	0.024	0.053	0.023	-0.042

* Data taken from Ref. [Jezuita, A.; Wieczorkiewicz, P.A.; Szatylowicz, H.; Krygowski, T.M. Solvent Effect on the Stability and Reverse Substituent Effect in Nitropurine Tautomers. *Symmetry* **2021**, *13*, 1223; doi:10.3390/sym13071223]

**Δ = FA – GP

Table S9. Relative energies (in kcal/mol) of C2-, C6-, C8-, N-NH_2 substituted purines.

		GP	Tol	Chf	o-Cr	THF	Py	EtOH	DMSO	H_2O	FA	Δ*	*average*
9H	**C2**	0.00	0.00	0.00	0.00	0.00	0.00	0.00	0.00	0.00	0.00	0.00	0.00
7H		3.59	2.54	1.81	1.51	1.44	1.12	0.90	0.77	0.71	0.69	2.91	1.51
3H		12.25	10.30	9.20	8.79	8.70	8.30	8.02	7.87	7.80	7.77	4.47	8.90
1H		15.92	12.46	10.35	9.53	9.35	8.52	7.93	7.62	7.47	7.41	8.51	9.66
9H	**C6**	0.00	0.00	0.00	0.00	0.00	0.00	0.00	0.00	0.00	0.00	0.00	0.00
7H		7.18	5.31	3.98	3.42	3.30	2.70	2.27	2.03	1.91	1.86	5.32	3.40
3H		7.09	6.00	5.43	5.22	5.18	4.98	4.84	4.77	4.74	4.72	2.36	5.30
1H		16.94	13.32	10.97	10.05	9.85	8.90	8.23	7.86	7.69	7.62	9.32	10.14
9H	**C8**	0.00	0.00	0.00	0.00	0.00	0.00	0.00	0.00	0.00	0.00	0.00	0.00
7H		2.62	1.41	0.57	0.23	0.15	-0.22	-0.48	-0.62	-0.69	-0.72	3.34	0.22
3H		2.95	3.14	3.29	3.35	3.36	3.42	3.47	3.49	3.50	3.51	0.55	3.35
1H		4.93	3.67	2.88	2.58	2.51	2.20	1.98	1.85	1.80	1.77	3.16	2.62
9H	**N-X∥**	0.00	0.00	0.00	0.00	0.00	0.00	0.00	0.00	0.00	0.00	0.00	0.00
7H		5.11	3.52	2.55	2.18	2.10	1.71	1.44	1.29	1.22	1.19	3.92	2.23
3H		5.66	4.80	4.33	4.16	4.12	3.94	3.82	3.76	3.73	3.72	1.94	4.20
1H		12.22	9.08	7.34	6.69	6.55	5.90	5.45	5.21	5.10	5.06	7.16	6.86
9H	**N-X⊥**	0.00	0.00	0.00	0.00	0.00	0.00	0.00	0.00	0.00	0.00	0.00	0.00
7H		4.98	3.35	2.33	1.93	1.84	1.43	1.14	0.98	0.90	0.87	4.10	1.98
3H		7.11	6.62	6.34	6.22	6.19	6.05	5.95	5.89	5.86	5.84	1.26	6.21
1H		13.03	10.09	8.39	7.75	7.61	6.96	6.50	6.26	6.14	6.09	6.94	7.88
range	**C2**	15.92	12.46	10.35	9.53	9.35	8.52	8.02	7.87	7.80	7.77		
	**C6**	16.94	13.32	10.97	10.05	9.85	8.90	8.23	7.86	7.69	7.62		
	**C8**	4.93	3.67	3.29	3.35	3.36	3.64	3.95	4.11	4.19	4.22		
	**N-X∥**	12.22	9.08	7.34	6.69	6.55	5.90	5.45	5.21	5.10	5.06		
	**N-X⊥**	13.03	10.09	8.39	7.75	7.61	6.96	6.50	6.26	6.14	6.09		

*Δ = FA – GP

Table S10. Solvation energies (in kcal/mol) of C2-, C6-, C8- and N-NH_2 coplanar substituted purines.

		GP	Tol	Chf	o-Cr	THF	Py	EtOH	DMSO	H_2O	FA
9H	**C2**	0.00	-3.99	-6.10	-6.86	-7.03	-7.78	-8.29	-8.57	-8.70	-8.75
7H		0.00	-5.04	-7.88	-8.95	-9.18	-10.25	-10.99	-11.39	-11.58	-11.66
3H		0.00	-5.93	-9.15	-10.32	-10.57	-11.73	-12.52	-12.94	-13.14	-13.22
1H		0.00	-7.44	-11.67	-13.25	-13.59	-15.18	-16.28	-16.87	-17.14	-17.26
9H	**C6**	0.00	-4.07	-6.19	-6.94	-7.11	-7.85	-8.35	-8.62	-8.74	-8.79
7H		0.00	-5.94	-9.39	-10.70	-10.99	-12.33	-13.27	-13.77	-14.01	-14.11
3H		0.00	-5.16	-7.85	-8.81	-9.01	-9.95	-10.59	-10.93	-11.09	-11.15
1H		0.00	-7.70	-12.15	-13.83	-14.19	-15.89	-17.06	-17.69	-17.99	-18.11
9H	**C8**	0.00	-4.95	-7.62	-8.59	-8.80	-9.77	-10.43	-10.78	-10.94	-11.01
7H		0.00	-6.16	-9.67	-10.98	-11.27	-12.60	-13.52	-14.02	-14.25	-14.35
3H		0.00	-4.76	-7.28	-8.19	-8.39	-9.30	-9.91	-10.24	-10.40	-10.46
1H		0.00	-6.22	-9.67	-10.94	-11.22	-12.50	-13.39	-13.86	-14.08	-14.18
9H	**N-X||**	0.00	-3.81	-5.87	-6.62	-6.78	-7.53	-8.04	-8.31	-8.44	-8.50
7H		0.00	-5.40	-8.42	-9.55	-9.79	-10.92	-11.71	-12.13	-12.33	-12.41
3H		0.00	-4.67	-7.19	-8.12	-8.32	-9.24	-9.87	-10.21	-10.37	-10.43
1H		0.00	-6.95	-10.75	-12.14	-12.45	-13.84	-14.80	-15.32	-15.56	-15.66
9H	**N-X⊥**	0.00	-3.48	-5.26	-5.90	-6.03	-6.66	-7.08	-7.31	-7.41	-7.46
7H		0.00	-5.10	-7.90	-8.94	-9.16	-10.20	-10.92	-11.30	-11.48	-11.56
3H		0.00	-3.97	-6.02	-6.79	-6.95	-7.71	-8.24	-8.53	-8.66	-8.72
1H		0.00	-6.42	-9.90	-11.18	-11.46	-12.73	-13.61	-14.08	-14.30	-14.40
range	**C2**	0.00	3.45	5.57	6.38	6.56	7.40	7.98	8.30	8.45	8.51
	**C6**	0.00	3.62	5.97	6.89	7.09	8.04	8.71	9.08	9.25	9.32
	**C8**	0.00	1.46	2.39	2.79	2.88	3.31	3.61	3.78	3.85	3.89
	**N-X||**	0.00	3.14	4.88	5.53	5.67	6.32	6.76	7.00	7.12	7.16
	**N-X⊥**	0.00	2.94	4.64	5.28	5.42	6.07	6.53	6.78	6.89	6.94

Table S11. Dipole moments, μ, (in D) of C2-, C6-, C8- and N-NH_2 substituted purines.

		GP	Tol	Chf	o-Cr	THF	Py	EtOH	DMSO	H_2O	FA	Δ*	average
9H	**C2**	3.20	3.72	4.03	4.15	4.17	4.29	4.38	4.43	4.45	4.46	*1.26*	*4.13*
7H		4.06	4.85	5.35	5.55	5.60	5.81	5.96	6.04	6.08	6.09	*2.04*	*5.54*
3H		5.98	7.44	8.24	8.53	8.60	8.90	9.10	9.22	9.27	9.29	*3.31*	*8.46*
1H		7.65	9.53	10.67	11.08	11.17	11.58	11.86	12.01	12.08	12.11	*4.46*	*10.98*
9H	**C6**	2.37	2.81	3.06	3.15	3.17	3.26	3.33	3.36	3.38	3.39	*1.01*	*3.13*
7H		6.79	8.19	9.06	9.41	9.49	9.87	10.12	10.25	10.31	10.34	*3.55*	*9.38*
3H		3.94	4.76	5.20	5.37	5.40	5.57	5.68	5.74	5.77	5.78	*1.84*	*5.32*
1H		8.22	10.27	11.43	11.86	11.95	12.39	12.71	12.88	12.96	13.00	*4.78*	*11.77*
9H	**C8**	5.44	6.63	7.31	7.57	7.63	7.89	8.07	8.16	8.20	8.22	*2.78*	*7.51*
7H		6.94	8.45	9.33	9.67	9.74	10.08	10.31	10.43	10.49	10.52	*3.57*	*9.60*
3H		3.04	3.56	3.87	3.99	4.01	4.14	4.23	4.28	4.30	4.31	*1.27*	*3.97*
1H		4.15	5.09	5.70	5.94	5.99	6.24	6.42	6.52	6.57	6.59	*2.44*	*5.92*
9H	**N-X‖**	3.95	4.66	5.05	5.20	5.23	5.38	5.49	5.54	5.57	5.58	*1.63*	*5.17*
7H		6.06	7.14	7.75	7.99	8.04	8.27	8.44	8.53	8.57	8.59	*2.53*	*7.94*
3H		4.59	5.48	5.98	6.17	6.21	6.40	6.53	6.60	6.63	6.64	*2.06*	*6.12*
1H		7.38	8.92	9.77	10.09	10.16	10.48	10.70	10.81	10.87	10.89	*3.51*	*10.01*
9H	**N-X⊥**	3.11	3.64	3.94	4.04	4.07	4.17	4.25	4.29	4.31	4.31	*1.21*	*4.01*
7H		6.21	7.27	7.85	8.06	8.11	8.33	8.48	8.56	8.60	8.61	*2.40*	*8.01*
3H		3.14	3.75	4.37	4.74	4.79	5.04	5.40	5.53	5.58	5.61	*2.46*	*4.80*
1H		7.03	8.43	9.20	9.48	9.54	9.82	10.02	10.12	10.17	10.19	*3.16*	*9.40*
range	**C2**	4.46	5.81	6.64	6.94	7.00	7.29	7.48	7.59	7.63	7.65		
	**C6**	5.84	7.46	8.37	8.71	8.79	9.13	9.38	9.52	9.58	9.61		
	**C8**	3.90	4.89	5.47	5.68	5.73	5.94	6.08	6.16	6.19	6.21		
	**N-X‖**	3.43	4.26	4.72	4.89	4.92	5.09	5.21	5.27	5.30	5.31		
	**N-X⊥**	3.93	4.79	5.26	5.44	5.48	5.65	5.77	5.84	5.87	5.88		

*Δ = FA – GP

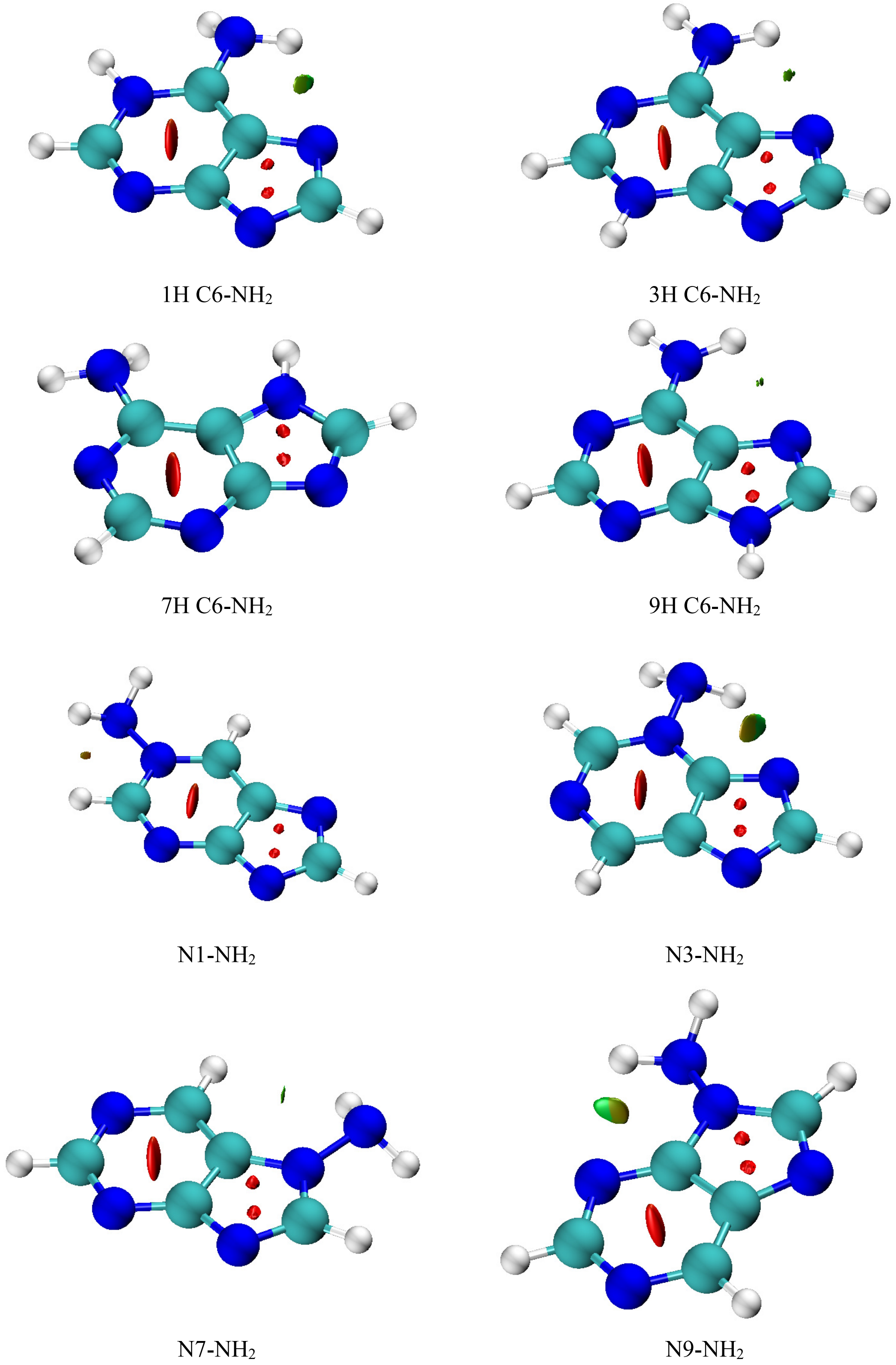

Figure S1. NCI analysis for selected systems in the gas phase. Visualization of reduced density gradient isosurfaces (isovalue = 0.5). Coloring of isosurfaces according to the type of interaction, by the value of sgn(λ_2)·ρ(r) (λ_2 – 2nd eigenvalue of the electron density Hessian matrix). Red – repulsive interactions, green – weak attractive interactions (van der Waals type).

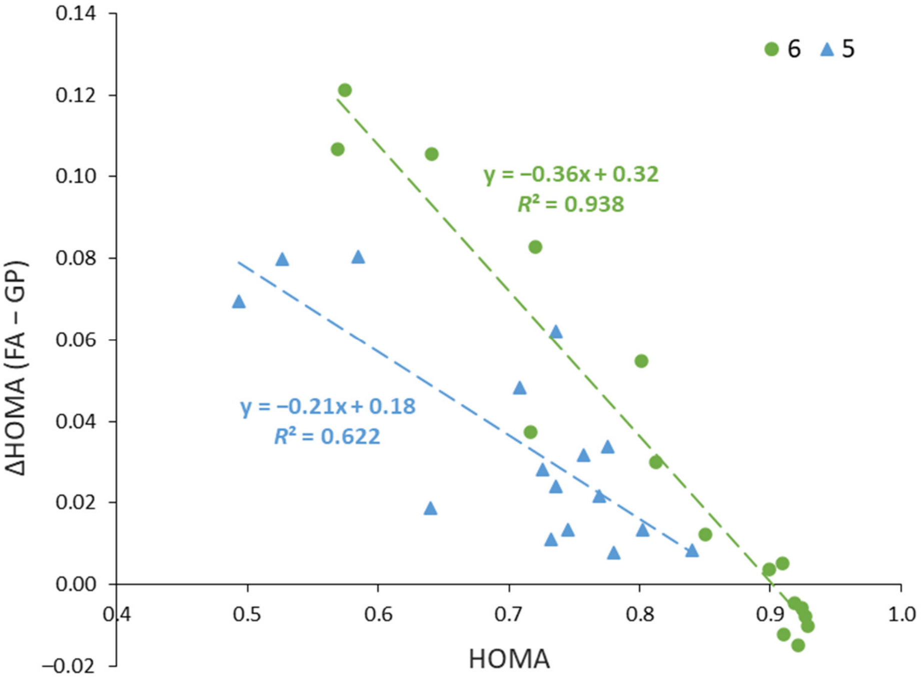

a)

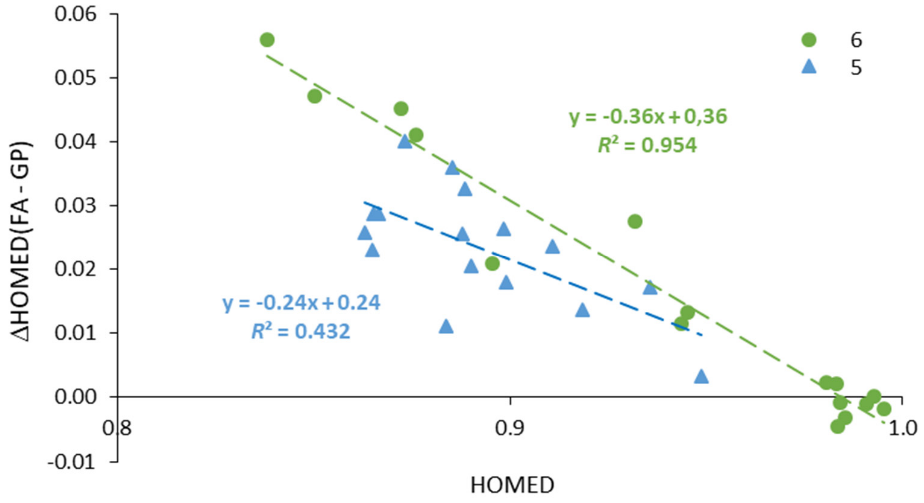

b)

Figure S2. Change in (a) HOMA and (b) HOMED of 6- and 5-membered rings between the most polar solvent, formamide (FA) and the gas phase plotted against the HOMA (HOMED) value for the respective ring of aminopurines in the gas phase.

Calculation of HOMED index in NH_2-substituted purine

$$\mathrm{HOMED} = 1 - \frac{1}{n}\sum_{i}^{n} \alpha_{\mathrm{j}}(R_{\mathrm{opt,j}} - R_{\mathrm{j,i}})^2$$

where n is the number of bonds taken into account when carrying out the summation, i means the type of bond (CC or CN), $R_{\mathrm{opt,j}}$ is the optimal length of a given bond (for CC and CN bonds $R_{\mathrm{opt\_CC}}$ = 1.4041 Å and $R_{\mathrm{opt\_CN}}$ = 1.3451 Å) assumed to be realized in fully aromatic systems with HOMED = 1, and $d_{\mathrm{j,i}}$ is an actual bond length in the studied system, α_{j} is an empirical normalization constant calculated for 6-membered ring ($\alpha_{\mathrm{\_CC}}$ = 96.43 and $\alpha_{\mathrm{\_CN}}$ = 96.89) and 5-membered ring ($\alpha_{\mathrm{\_CC}}$ = 85.77 and $\alpha_{\mathrm{\_CN}}$ = 86.79) according with Eqs. from Ref. [Raczyńska, E.D.; Hallman, M.; Kolczyńska, K.; Stępniewski, T. On the Harmonic Oscillator Model of Electron Delocalization (HOMED) Index and its Application to Heteroatomic π-Electron Systems. *Symmetry* **2010**, *2*, 1485–1509, doi: 10.3390/sym2031485]:

for 6R: $\alpha = 2 \cdot \{(R_{opt} - R_s)^2 + (R_{opt} - R_d)^2\}^{-1}$

for 5R: $\alpha = (2i + 1) \cdot \{(i + 1) \cdot (R_{opt} - R_s)^2 + i \cdot (R_{opt} - R_d)^2\}^{-1}$

where R_s is the reference single CC, CN bond lengths calculated for ethane and methylamine, respectively ($R_{\mathrm{s\_CC}}$ = 1.5338 Å and $R_{\mathrm{s\_CN}}$ = 1.4729 Å), R_d is the reference double CC, CN bond lengths calculated for ethene and methylimine, respectively ($R_{\mathrm{d\_CC}}$ = 1.3415 Å and $R_{\mathrm{d\_CN}}$ = 1.2794 Å).

All parameters were calculated using B97D3/AUG-cc-pVDZ computational level.

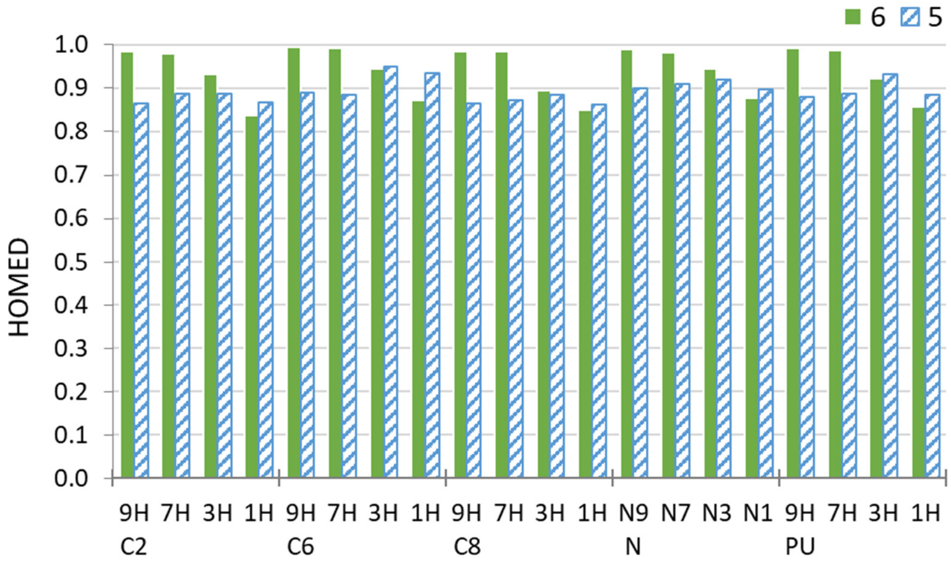

Figure S3. Values of HOMED index for 6- and 5-membered rings of C2, C6, C8 and N amino-substituted derivatives of purine in four tautomeric forms, calculated for the gas phase geometry. PU are the values for unsubstituted purine.

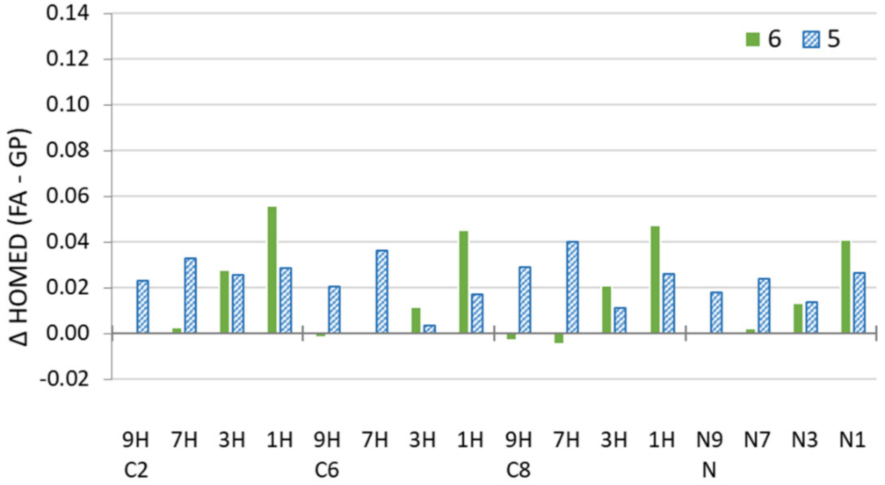

a)

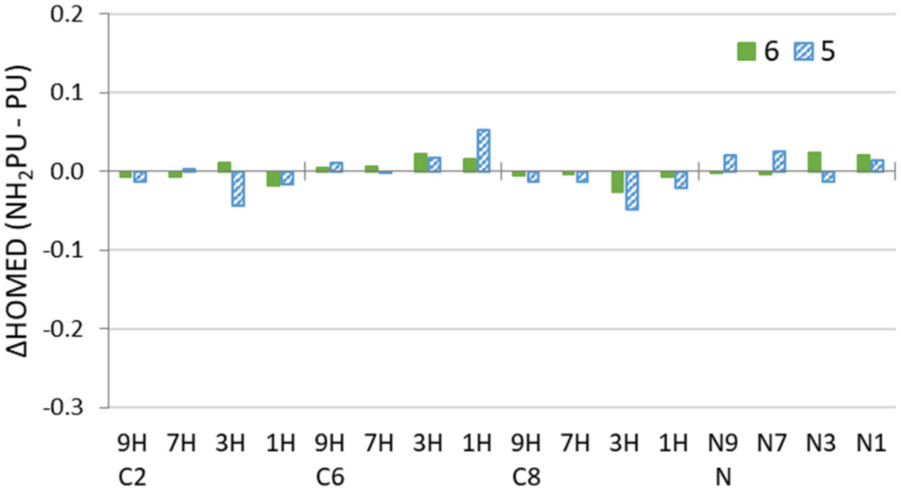

b)

Figure S4. Changes in HOMED index due to (a) solvation ΔHOMED (FA – GP), calculated by subtracting the value in the gas phase from the value in the most polar solvent considered, formamide, and (b) substitution, ΔHOMED, calculated by subtracting the value for unsubstituted purine (PU) from the value for substituted purine (NH_2PU).

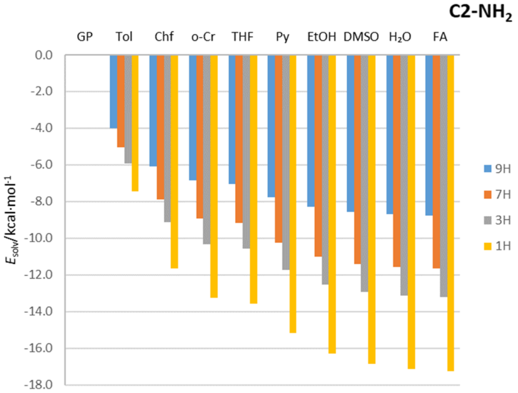

(a)

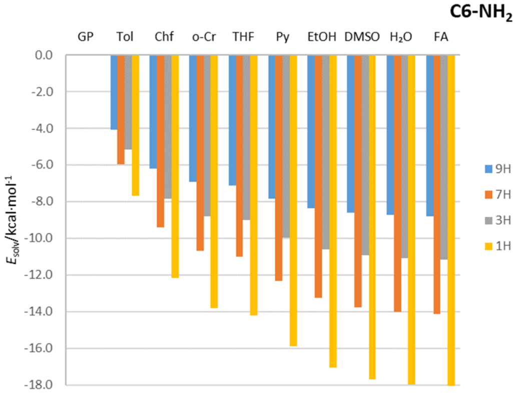

(b)

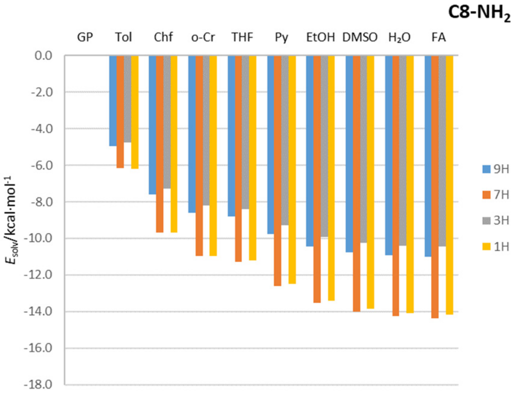

(c)

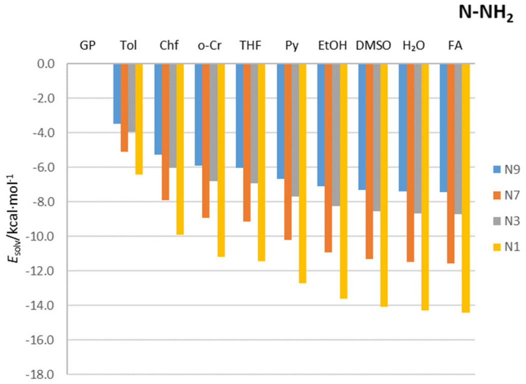

(d)

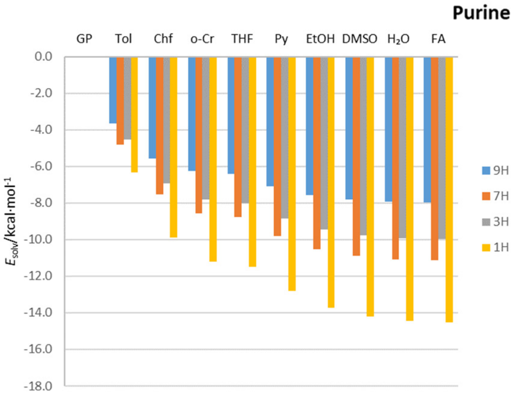

(e)

Figure S5. Solvation energies of tautomers, E_{solv}, for (a) C2-NH$_2$, (b) C6-NH$_2$ (c) C8-NH$_2$ (d) N-NH$_2$ purine derivatives, and (e) unsubstituted purines.
